# S6P mutation in Delta and Omicron variant spike protein significantly enhances the efficacy of mRNA COVID-19 vaccines

**DOI:** 10.3389/fimmu.2024.1495561

**Published:** 2025-01-03

**Authors:** Yong-Sik Bong, David Brown, Ezra Chung, Neeti Ananthaswamy, Renxiang Chen, Evan Lewoczko, William Sabbers, Athéna C. Patterson-Orazem, Zachary Dorsey, Yiqing Zou, Xue Yu, Jiening Liang, Jiaxi He, Steven Long, Dong Shen

**Affiliations:** ^1^ RNAimmune, Inc., Germantown, MD, United States; ^2^ Guangzhou RNAimmune, Ltd., Guangzhou, China

**Keywords:** lipid nanoparticles (LNPs), mRNA vaccines, SARS-CoV-2 spike protein, immunogenicity, neutralizing antibodies, Delta and Omicron variant, RV-1730 and RV-1731 vaccines

## Abstract

**Background:**

The unrelenting emergence of SARS-CoV-2 variants has significantly challenged the efficacy of existing COVID-19 vaccines. Enhancing the stability and immunogenicity of the spike protein is critical for improving vaccine performance and addressing variant-driven immune evasion.

**Methods:**

We developed an mRNA-based vaccine, RV-1730, encoding the Delta variant spike protein with the S6P mutation to enhance stability and immunogenicity. The vaccine’s immunogenicity and protective efficacy were evaluated in preclinical models, including monovalent (RV-1730) and bivalent (RV-1731) formulations targeting the Delta and BA.1 variants. Additionally, the effectiveness of RV-1730 as a heterologous booster following primary vaccination with BNT162b2 (Pfizer-BioNTech) and mRNA-1273 (Moderna-NIAID) was assessed.

**Results:**

RV-1730 elicited significantly stronger B and T cell responses and more durable neutralizing antibodies compared to S2P-based vaccines. The bivalent RV-1731 vaccine demonstrated broad neutralizing activity against emerging variants, including XBB1.5 and JN.1. Importantly, RV-1730, when used as a heterologous booster following initial immunization with BNT162b2 or mRNA-1273, significantly enhanced neutralizing antibody titers against multiple variants, including Delta and Omicron. Both RV-1730 and RV-1731 provided superior protection in preclinical models, indicating enhanced efficacy due to the S6P mutation.

**Conclusion:**

The incorporation of the S6P mutation into the Delta variant spike protein significantly enhances the immunogenicity and efficacy of mRNA-based COVID-19 vaccines. The strong performance of RV-1730 as a heterologous booster and the broad-spectrum activity of the bivalent RV-1731 vaccine underscore their potential as versatile and effective vaccination strategies against SARS-CoV-2 and its evolving variants.

## Introduction

The novel coronavirus, SARS-CoV-2, emerged in 2019, leading to a global pandemic with profound public health implications (1). By November 2022, there were over 628 million confirmed cases and 8 million deaths worldwide. As a betacoronavirus, SARS-CoV-2 affects both the upper and lower respiratory tracts, though severe cases often involve the lower respiratory tract, particularly in the elderly and individuals with pre-existing conditions. The tropism of the virus can vary depending on the viral strain or variant (2, 3). In response to this global crisis, vaccines such as SPIKEVAX (Moderna-NIAID) and COMIRNATY (Pfizer-BioNTech) were rapidly developed and deployed, proving instrumental in mitigating the spread of the virus and reducing disease severity (4–7). However, the emergence of new variants of concern (VOCs), including the Delta variant identified in 2020, posed significant challenges to the efficacy of these vaccines (8, 9). Variants like Delta, which exhibited higher transmissibility and partial immune evasion, marked key phases in the pandemic, though newer variants have now taken precedence. The subsequent emergence of the Omicron variant (B.1.1.529) in late 2021 (10), with over 30 mutations in the spike protein and even greater resistance to neutralizing antibodies (10–15), further underscored the need for vaccine innovations that could address these evolving threats.

The spike (S) protein of SARS-CoV-2, essential for viral entry into host cells (16), is a trimeric class I fusion protein that exists in a metastable prefusion conformation (17–19). This conformation is crucial for effective antibody-mediated neutralization (20), making the S protein a primary target for vaccine development. However, the prefusion state is inherently unstable, posing a significant challenge in vaccine design. To overcome this, the initial mRNA vaccines utilized the S2P mutation, which introduced two proline substitutions (KV986PP) that effectively stabilized the S protein in its prefusion form (19, 21, 22). Both Moderna-NIAID and Pfizer-BioNTech vaccines employed this S2P-stabilized spike protein, contributing to their success in early vaccine rollouts (23–26). Researchers have advanced this foundational work by developing a superior stabilization method involving the S6P mutation. This includes the original KV986PP mutation, along with four additional proline substitutions at positions F817P, A892P, A899P, and A942P, which further stabilize the spike protein in its prefusion form (19, 27). This design not only further stabilizes the prefusion conformation but also enhances the expression and thermal stability of the spike protein, leading to more robust immunogenicity (21, 28, 29). Comparative analyses of AlphaFold structural models of wild-type (WT), Delta, and BA.1 spike proteins, with and without stabilizing mutations, suggest that the Delta and BA.1 variants can incorporate the S6P mutations, which enhance prefusion stability and expression levels similarly to the WT spike protein. This makes the S6P-modified proteins more promising candidates for next-generation vaccines (see **Supplementary Figure 1**). In this study, we present RV-1730, an mRNA-based vaccine encoding the Delta variant spike protein incorporating the S6P mutation, delivered via lipid nanoparticles (LNPs). Additionally, we introduce RV-1731, a bivalent mRNA vaccine designed to target both the Delta and BA.1 variants. The primary focus of this research is the evaluation of RV-1730 as a monovalent mRNA vaccine targeting the Delta variant, assessing its immunogenicity and protective efficacy. RV-1731 is subsequently examined for its potential broader applicability, particularly in addressing emerging variants such as XBB1.5 and JN.1.These vaccines were designed not only to enhance stability and immunogenicity through the S6P mutation but also to incorporate additional modifications, such as the R682S and R685G mutations, to prevent furin cleavage, and variant-specific mutations like T19R, L452R, and D614G to further optimize antigen expression. Beyond their primary use, the efficacy of these vaccines as heterologous boosters—following initial vaccination with commercial vaccines like BNT162b2 (Pfizer-BioNTech) and mRNA-1273 (Moderna-NIAID)—was evaluated. This approach was particularly relevant given the ongoing need to improve immune responses against evolving variants. Our findings indicate that RV-1730, when used as a heterologous booster, significantly enhances neutralizing antibody titers against multiple SARS-CoV-2 variants, including Delta and Omicron. Furthermore, the bivalent RV-1731 demonstrated broad neutralizing activity and robust T cell responses, positioning it as a strong candidate for booster immunization strategies. Overall, the S6P mutation within the Delta variant spike protein provides a critical enhancement in the stability and efficacy of mRNA-based COVID-19 vaccines. RV-1730 and RV-1731 not only offer superior protection compared to S2P-based vaccines but also serve as potent boosters that can effectively combat the challenges posed by new and emerging variants. These findings highlight the importance of continued innovation in vaccine design to ensure broad-spectrum immunity against SARS-CoV-2.

## Materials and methods

### Vaccines

All reference vaccines, including mRNA-1273 (monovalent and bivalent with original and Omicron BA.4/BA.5) and BNT162b2 (monovalent and bivalent with original and Omicron BA.4/BA.5), were purchased from RefDrug, Inc. The RV-1730 monovalent and RV-1731 bivalent vaccines were constructed and formulated by RNAimmune, Inc. RV-1770 and RV-1771 are mRNA vaccines developed by RNAimmune to specifically target the Delta and Omicron BA.1 SARS-CoV-2 variants, respectively. Both vaccines include the S2P mutations (two proline substitutions, KV986PP) to stabilize the spike protein in its prefusion form, a feature also present in commercial mRNA vaccines such asModerna-NIAID’s and Pfizer-BioNTech’s. Additionally, RV-1770 and RV-1771 incorporate S6P mutations (four extra proline substitutions at F817P, A892P, A899P, and A942P), providing enhanced structural stability to the spike protein for Delta and Omicron BA.1, respectively.

### Cell culture

HEK293T cells were purchased from American Type Culture Collection (ATCC, Manassas, VA). HEK293T cells were grown in Dulbecco’s modified Eagle’s medium (DMEM; Life Technologies) supplemented with 10% fetal bovine serum (FBS).

### Animals

Specific-pathogen-free (SPF) Balb/c mice and female K18-hACE2 transgenic mice (B6.Cg-Tg(K18-ACE2)2Prlmn/J), aged 6 to 8 weeks, were obtained from Jackson Laboratories (Bar Harbor, ME) and used in this study. All mouse experiments were conducted in AIC Biotech (Rockville, MD), Denovo Biotechnology (Ijamsville, MD), and RNAimmune (Germantown, MD) in accordance with the regulations of the Institutional Animal Care and Use Committee (IACUC).

### Biosafety

All experiments with infectious SARS-CoV-2 were conducted under biosafety level 3 (BSL3) or animal BSL3 (ABSL3) at The George Mason University using standard operating procedures and were approved by the BSL3 Advisory Group, the Institutional Biosafety Committee (IBC), and the Institutional Animal Care and Use Committee (IACUC).

### Animal experiments

#### Immunization studies

All animal procedures were conducted by AIC Biotech (Rockville, MD, USA), Denovo Biotechnology (Ijamsville, MD, USA), and RNAimmune (Germantown, MD, USA) in compliance with the guidelines set by the Institutional Animal Care and Use Committee (IACUC). Naïve 6- to 8-week-old female Balb/c mice (The Jackson Laboratory) were randomly divided into groups of five – eight mice and vaccinated via intramuscular injection with RV-1730 (1, 5, 10 and 20 μg per mouse in 50 μl). All mice were immunized twice at 21-day intervals. Blood was taken before each immunization at 14 and 35 days after the first immunization via submandibular bleeding. The blood was allowed to clot overnight at 4°C before the serum was harvested by centrifugation at 10,000 xg for 10 minutes at 4°C. Samples were stored at 4°C until further analysis. For homologous and heterologous as well as booster administration, female Balb/c mice (The Jackson Laboratory) received intramuscular injections of 5 µg each of BNT162b2, mRNA-1273, and RV-1730 on days 0 and 21 for as a prime-boost vaccination. To standardize dosing across different vaccine sources, mRNA concentration measurements were conducted for BNT162b2, mRNA-1273, and RV-1730. This ensured comparable dose levels, with adjustments made for any differences in mRNA content to align with the commercial vaccines. Then, a booster dose of 2.5 µg for each vaccine was administered on day 72 post the first immunization. Blood samples were collected at 14, 35, 49, 63, 77, and 105 days after the initial immunization. Spleens were harvested at day 105 for T cell immunity experiments.

#### Live virus challenges

Female K18-hACE2 mice (B6.Cg-Tg(K18-ACE2) Primn/J, The Jackson Laboratory), aged 6-8 weeks, underwent immunization on day 0 and day 14 at Nobel Life Science (NLS) in Sykesville, MD, USA in compliance with the guidelines set by the IACUC. On day 21, the mice were transported to George Mason University (GMU; Manassas, VA, USA) and acclimated for 7 days in ABSL-2 containment. Blood samples were collected on day 27 at GMU to prepare serum before virus challenge. Serum aliquots from day 13 and day 27 were sent to the sponsor for further analysis. On day 28, the mice were transferred to ABSL-3 containment and intranasally challenged with either 5.5 x 10^4^ PFU/mouse of SARS-CoV-2/human/ITA/INMI1/2020 (wild type) or 1.5 x 10^4^ PFU/mouse of SARS-CoV-2 B.1.1617.2 Delta variant. Daily monitoring for mortality, body weight, body temperature, and signs of distress was conducted for 13 days (wild type) or 14 days (Delta) post-challenge. Surviving animals were euthanized 13-14 days after the challenge.

### *In vitro* transcription (IVT)

The optimal conditions for *in vitro* transcription (IVT) were determined, and the IVT process was carried out on the mRNA-1730 producing plasmid. Initially, the plasmid was linearized using XhoI and purified. The mRNA was then synthesized through the following steps: RNase-free water and NTPs were added to a reaction tube. CleanCap AG (TriLink) was added to the tube and vortexed for thorough mixing. The liquid was briefly spun down to collect. 10X Transcription Buffer was added, and the mixture was vortexed again. The liquid was briefly spun down. DNA template was added to the reaction. Enzymes were added to the reaction. The mixture was well-mixed by flicking or inverting the tube 10 times, and the liquid was briefly spun down. The reaction was incubated at 37°C for 4 hours. The components of the 10X Transcription Buffer consisted of 400 mM Tris-HCl (pH 8), 100 mM DTT, 20 mM Spermidine, 0.02% Triton X-100, 165 mM Magnesium Acetate, and DNase/RNase-Free Water. In the final reaction, the concentrations were adjusted to 1X Transcription Buffer, 5 mM ATP, 5 mM CTP, 5 mM GTP, 5 mM N-1metyl-pseudoUTP, 4 mM CleanCap AG, Murine RNase Inhibitor (1 unit/μl), *E. coli* Inorganic Pyrophosphatase (0.002 units/μl), T7 RNA Polymerase (8 units/μl), and 25-50 ng/μl linear DNA.

### RV-1730 and RV-1731 formulation

RV-1730 and RV-1731 are lipid nanoparticles (LNPs) carrying mRNA encoding the prefusion stabilized spike protein (S6P) of SARS-CoV-2. mRNA-LNPs were generated by using the NanoAssemblr Ignite (Precision NanoSystems). The formulated LNPs were collected in a labeled tube. After motor repositioning, the lid was opened safely, and the tube labeled “mRNA-LNP” was removed for immediate characterization. For LNP characterization, 25-50 μl of the sample fraction was mixed with ultra-pure water and particle size was measured on Zeta Sizer (Malvern Pananalytical). The sample was transferred to a dialysis cassette and dialyzed overnight. The LNP formulations were concentrated using ultra-centrifugal filters, filtered through a 0.22-μm filter, and the final mRNA concentration and encapsulation efficiency were measured using the Quant-it Ribogreen Assay Kit (Invitrogen). LNPs were stored at 4°C (in PBS) or -20°C.

### Western blot

Lysates from transfected HEK293T cells were prepared in 200 μl RIPA buffer (Thermo Scientific) with a proteinase inhibitor cocktail in a 6-well plate (one well per sample). The plate was then placed on ice for 30 minutes and gently mixed. Subsequently, the cell lysates were transferred to 1.5 ml tubes, briefly sonicated, and centrifuged for 15 minutes at maximum speed. BCA assays were employed to determine protein concentrations in the lysates. For gel electrophoresis, inactivated cell lysates (10-20 μg per lane) were heated at 95°C for 5 minutes, separated on an SDS-PAGE gel (4-12% NuPAGE, Invitrogen) with MES Running buffer for 30 minutes at 200 V, and transferred onto a PVDF membrane using the iBlot2 system (Invitrogen). Following blocking with 5% milk/TBST (1x Tris-buffered saline containing 0.1% Tween-20), the membranes were incubated with primary antibodies in 5% milk/TBST at 4°C overnight. The primary anti-S monoclonal antibodies for S1, S2, and RBD were purchased from Invitrogen (S1: MA5-36249; S2: MA5-36254; RBD: MA5-36253, respectively). After washing with PBST, secondary antibodies conjugated with HRP were applied to the membrane in 5% milk/TBST for 1 hour at room temperature. Chemiluminescence for image acquisition was achieved using Clarity Western ECL substrate (Bio-Rad), and band intensities were quantified using an Azure ChemiDoc Imaging System (Azure Biosystem).

### ELISA

MaxiSorp plates (BioLegend) were coated with 100 μl of recombinant S1 or RBD (1 μg/ml) in sodium bicarbonate buffer overnight at 4°C. The antigens used for coating included S1 proteins for multiple SARS-CoV-2 variants: Alpha (Sino Biological, Cat #40591-V08H12), Beta (Sino Biological, Cat #40591-V08H15), Gamma (Sino Biological, Cat #40589-V08H26), Delta (Sino Biological, Cat #40150-V08B1), BA.1 (Sino Biological, Cat #40591-V08H41), and BA.2 (Sino Biological, Cat #40589-V08H28). Additionally, RBD proteins were used for the Wild Type (Sino Biological, Cat #40592-V08H), Alpha (Sino Biological, Cat #40592-V08H82), Beta (Sino Biological, Cat #40592-V08H85), Delta (eEnzyme, Cat #SCV2-RBD-IN2P), BA.1 (Sino Biological, Cat #40592-V08H121), BA.2 (Sino Biological, Cat #40592-V08H123) and XBB.1.5 (Sino Biological Cat # 40591-V08H47).The wells were washed 3 times with PBS-T and incubated the plates with 200 μl of blocking buffer for 2 hours at room temperature (RT). After washing 3 times with PBS-T, the plate was incubated with 100 μl of sera for 2 hours at RT. And the plate washed 5 times with PBS-T and incubated with HRP-conjugated secondary antibody for 1 hour at RT. Finally, the plate was washed 5 times with PBS-T and incubated the plates with 100 μl of 1x TMB substrate for 10 minutes. The reaction was stopped by adding 100 μl of 1N-HCl and read at 450 nm using the Cytation7. For reciprocal endpoint IgG titers, 2-fold serial diluted sera were added to the wells immobilized with different spike proteins. All procedures were the same as described above. For isotyping of binding antibodies, MaxiSorp plates (BioLegend) were coated with 100 μl of recombinant S1 protein (1 μg/ml) in sodium carbonate buffer and left to incubate overnight at 4°C. The plates were then washed three times with PBS-T and blocked with 200 μl of blocking buffer for 2 hours at room temperature (RT). Following another three PBS-T washes, 100 μl of sera was added and incubated for 2 hours at RT. After five additional PBS-T washes, plates were incubated with HRP-conjugated anti-mouse IgG, IgG1, IgG2a, or IgG2c (Abcam) for 1 hour at RT. Following five more PBS-T washes, 100 μl of 1x TMB substrate was added for 10 minutes. The reaction was stopped by adding 100 μl of 1N-HCl, and optical density was read at 450 nm using the Cytation7.

### Flow cytometry

To evaluate the expression of spike proteins on HEK293T cells transfected with mRNA and mRNA-LNP, flow cytometry was performed. The culture media was removed from the transfected cells by aspiration and the cells were washed with 1x PBS. The cells were detached by adding 300 μl of 0.05% Trypsin-EDTA, incubated for 5 minutes at 37°C, 5% CO_2_ incubator and counted in 1 ml of complete media. 2 x 10^5^ cells were aliquoted in a 96 round bottom well plate and centrifuged for 5 minutes at 1,800 rpm to pellet down the cells. The cells were washed twice with 1x PBS and twice with FACS Cell Staining Buffer. Cells were resuspended in 100 μl of staining buffer containing primary antibody at a concentration of 5 μg/ml at 4°C for 1 hour. After the cells were washed 2 times with FACS Staining buffer, they were resuspended in 100 μl of staining buffer containing 1:200 diluted anti-Rabbit IgG(H+L)-FITC for Clone H4 and anti-FLAG M2-FITC for hACE2 (5 μg/ml) at 4°C for 30 minutes, then washed twice with FACS staining buffer. The stained cells were resuspended in 200 μl of PBS containing 100 ng/ml of DAPI (BD Pharmingen, 564907). Flow cytometry was performed using a BD FACSCelesta for data acquisition and a FlowJo V10.7.2 for data analysis.

### ELISpot

Spleens were harvested from mice and processed into single-cell suspensions in RPMI1640 media supplemented with 10% heat-inactivated fetal calf serum and penicillin/streptomycin (R10 media). To eliminate red blood cells, RBC lysis buffer (KD Medical) was utilized, and the cells were subsequently resuspended in R10 media to terminate the lysis process. Following cell counting, 200,000 cells per well were seeded into 6-well plates, which were then utilized for the Mouse IFN-γ ELISpot PLUS (HRP) kit (Mabtech). The cells were stimulated for 16 hours at 37°C with 2 μg of PepMix™ SARS-CoV-2 (Spike B.1.617.2/Delta) from JPT Peptide Technologies GmbH, while media alone served as a negative control. Spots were developed following the manufacturer’s instructions, and their quantification was performed using Cytation7.

### Intracellular cytokine staining

The splenocytes were counted, and 1 x 10^6^ cells per well were seeded into 96-well plates. Subsequently, the cells were stimulated in the presence of a protein transport inhibitor (Brefeldin A, Biolegend) for 16 hours at 37°C with 2 μg of PepMix™ SARS-CoV-2 (Spike B.1.617.2/Delta) from JPT Peptide Technologies GmbH, while media alone served as a negative control. After stimulation, Splenocytes were stained as follows; Centrifuge the plate at 400 x g for 8 minutes at 4°C, then carefully resuspend the cell pellets in the remaining fluid. Wash the cells twice with azide- and serum/protein-free PBS, ensuring the supernatant is completely decanted. Add 100 µL of the Fixable Viability Dye (FVD-V450) solution (1:1000 dilution in PBS) to each well and mix immediately by pipetting. Incubate for 30 minutes at 2–8°C. Wash the cells twice with Cell Staining Buffer. Block non-specific Fc-mediated interactions by incubating the cells with 0.5 µg of anti-Mouse CD16/CD32 antibody in 50 µL of Cell Staining Buffer for 10 minutes at 2–8°C. Add 50 µL of primary antibody mixtures (anti-CD3-FITC, CD4-PerCP-Cy5.5, and CD8-BV650, Biolegend) and incubate for 30 minutes at 2–8°C. Wash cells twice with Cell Staining Buffer. After the last wash, discard the supernatant. Fix the cells by adding 200 µL of IC Fixation Buffer to each well. Incubate for 60 minutes at room temperature. Centrifuge at 400–600 x g for 5 minutes at room temperature and discard the supernatant. Add 200 µL of 1X Permeabilization Buffer to each well and centrifuge at 400–600 x g for 5 minutes at room temperature. Discard the supernatant. Resuspend the pellet in residual volume and adjust to approximately 100 µL with 1X Permeabilization Buffer. Block with 2% normal mouse/rat serum by adding 2 µL directly to the cells and incubate at room temperature for 15 minutes. Without washing, add the recommended amount of directly conjugated antibody mix (anti-IFN-γ-PE, IL-4-APC, Biolegend) for detection of intracellular antigen(s) and incubate for at least 30 minutes at room temperature. Centrifuge at 400–600 x g for 5 minutes at room temperature and discard the supernatant. Wash cells with 200 µL of 1X Permeabilization Buffer and centrifuge again. Resuspend stained cells in 200 µL of PBS. Perform flow cytometry (FACS) analysis. Ensure compensation for spillovers when multiple fluorophore-conjugated antibodies are used.

### Quantification of intracellular cytokine production

The splenocytes were counted, and 2 x 10^5^ cells per well were seeded into 96-well plates. Subsequently, the cells were stimulated for 72 hours at 37°C with 2 μg of PepMix™ SARS-CoV-2 (Spike B.1.617.2/Delta) from JPT Peptide Technologies GmbH, while media alone served as a negative control. Following stimulation, the plates were centrifuged, and the supernatant was collected and frozen at -80°C for cytokine detection. The analysis of secreted cytokines was conducted using a murine 11-plex kit through a multiplex bead-based technology (Luminex) assay, employing a Luminex100/200 instrument (Luminex). Supernatants were subjected to a 2-fold dilution before measurements and analyses to assess the levels of various cytokines.

### Pseudovirus neutralizing assays

#### Generation of SARS-CoV-2-pseudovirus particles

Murine Leukemia Virus (MLV) particles pseudotyped with a SARS-CoV-2 Spike protein construct were generated in HEK293T (ATCC) cells. All the plasmid DNAs were purified with ZymoPURE II Plasmid Midiprep Kit (Zymo Research). In brief, 8 million HEK293T cells were plated into a 10-cm tissue culture dish (Santa Cruz) in 16 ml DMEM (Genesee Scientific) containing 10% FBS (Corning Life Sciences) without any antibiotics. The following day, the cells were transfected with 8 µg pTG-Luc, 6 µg pCMV-MLVgag-pol and 6 µg pcDNA3.1-SARSCoV-2-SpikeΔC19 of different variants using Lipofectamine 3000 reagent (Thermo Fisher). The cells were cultured for an additional 48 hours. The supernatant was collected into a 50-ml Falcon tube and spun at 290 ×g for 7 minutes. The supernatant (pseudotyped virus solution) was then passed through a 0.45 μm filter (Santa Cruz) using appropriate syringe. The pseudotyped virus solution was then aliquoted into cryovials and stored at -80°C. Each 10-cm cell culture dish produces about 16 ml SARS-CoV-2-PP. The SARS-CoV-2-PP was tested for the quality control with HEK293-ACE2 cell line (created at Codex BioSolutions).

#### Neutralizing assay with pseudovirus

Due to limited serum availability, a pooled serum sample was prepared for each group and then serially diluted threefold in quadruplicate. It was heat inactivated by incubating the serum at 56°C for 30 minutes. The day before the infection, 7.5 x 10^3^ HEK293-ACE2 cells were plated into a 384-well white clear plate (Corning Life Sciences) precoated with Poly D Lysine (Trevigen) in 15 µl culture medium (DMEM containing 10% Fetal Clone II Serum, Fisher Scientific). The cell plate was placed in a CO_2_ incubator at 37°C. On the 2nd day, the serum to be tested was diluted in the culture medium on a 96-well compound plate. They are 5X of the final concentrations. 65 µl of SARSCoV-2 MLV pseudoviruse particles (pp) were mixed with 26 µl of the testing sample prepared above and incubated at 37°C for 1 hour. After the medium in each well of 384-well cell plate was removed, 17.5 µl of each serum-pp mixture was added into each well. The plate was centrifuged at 54 xg for 15 minutes at 4°C and additional 7.5 µl of the culture medium was then added into each well. Luciferase activities were measured with Firefly Luciferase Assay Kit (Codex BioSolutions Inc). IC_50_ values were calculated based on curve fitting in GraphPad Prism (data was normalized as the percentage of infectivity).

### Plaque reduction neutralization (PRNT) assay

The neutralizing titers of serum specimens collected on study day -1, day 13 and day 27 were assessed using plaque reduction neutralization test (PRNT). All sera were heat inactivated by incubation at 56°C for 30 minutes prior to testing. Six serial two-fold dilutions (1/2 to 1/64) of each serum were prepared in EMEM and 25 μl of each dilution was combined with 25 μl of SARS-CoV-2/human/ITA/INMI1/2020 viral stock, mixed and incubated at 37°C for 1 hour. Supernatant was removed from Vero-E6 cells seeded the night before in 12 well plates and replaced with 300 μl of Eagle Minimum Essential Medium (EMEM) in each well. The incubated virus + diluted serum mixtures were added on top of Vero E6 monolayer, and plates were incubated at 37°C/5% CO_2_ for 1 hour. Plates were shaken every 10-15 minutes during incubation. After one-hour incubation, 1.5 ml overlayer containing 2X EMEM and 0.6% agarose at a ratio of 1:1 was added to the plates. Plates were incubated for 72 hours at 37°C/5% CO_2_ after solidification of agarose at room temperature. Following incubation, cells were fixed by adding 0.5ml of 10% formaldehyde into each well on top of the agarose and incubating at room temperature overnight. The palettes of agarose were gently removed, and the cellular monolayers were stained for 10 to 15 minutes with the addition of 0.5 ml of 1% crystal violet. After staining, the plates were washed with water several times to remove excess stain solution and plaques were manually enumerated for each viral dilution. The neutralizing titers were calculated as the reciprocal of the lowest dilution that resulted in a greater than 50% reduction (PRNT_50_) or 90% reduction (PRNT_90_) in PFU relative to negative control sera. Human sera collected from two fully vaccinated males and serum from naïve mice were used as a positive and negative controls, respectively.

### Histology

A portion of right lung tissue of each mouse euthanized was collected and placed into 10% formalin. Lung tissue samples fixed with formalin were removed from ABSL3 and shipped to Histoserv (Germantown, MD) for histopathological assays.

### Statistical analysis

The data were presented as the mean ± standard deviation (SD). Statistical analyses were carried out using GraphPad Prism 10 (GraphPad Software, La Jolla, CA). Group differences were assessed using Student’s t-test, one-way ANOVA with Dunnett’s multiple comparisons test and two-way ANOVA with Tukey’s multiple comparisons test. A significance level of P < 0.05 was deemed statistically significant.

## Results

### Delta S6P mRNA is expressed at a significantly higher level than S2P in the LNP system

**Figure 1:**
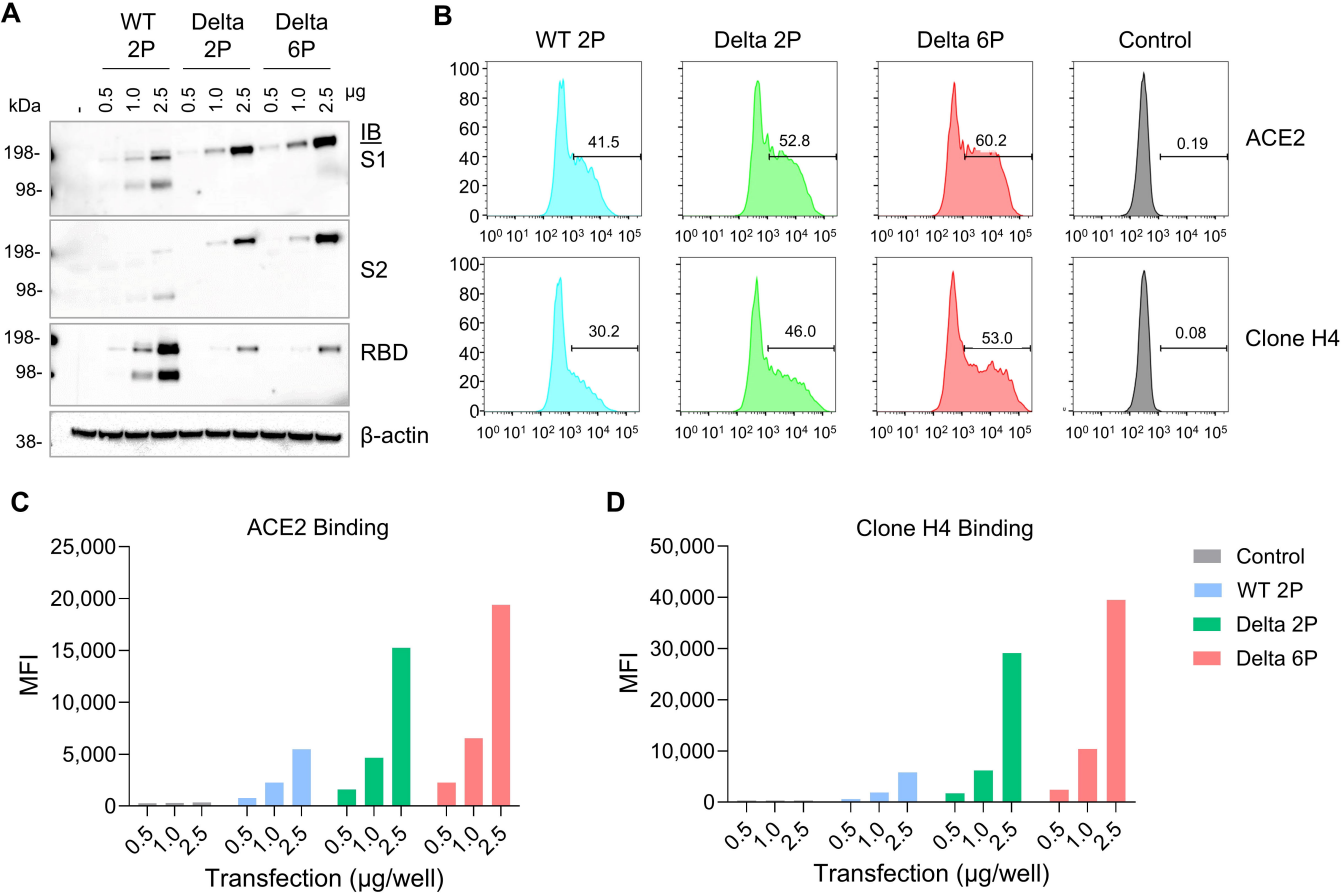
*In vitro* expression of mRNA encoding Spike protein with 2P or 6P mutations in various strains. **(A)** HEK293T cells were transfected with mRNA encoding different forms of the SARS-CoV-2 S protein. After 48 hours, cell lysates were analyzed by Western Blot using an anti-SARS-CoV-2 S2-specific antibody. **(B–D)** the cells underwent flow cytometry to measure surface expression, with staining performed using a Flag-tagged ACE2 receptor and the anti-S antibody clone H4 **(B)** The mean fluorescence intensity (MFI) was calculated to assess the cell surface expression levels, using the anti-Flag antibody **(C)** and the anti-S antibody clone H4 **(D)**.

Initially, we evaluated the expression of the SARS-CoV-2 Spike S6P antigen in HEK293T cells post-transfection with custom-designed mRNA targeting various viral variants. Within the HEK293T cell system, we investigated the binding specificity and affinity of the SARS-CoV-2 spike protein with human ACE2 (hACE2). Different forms of SARS-CoV-2 mRNAs, including S WT S2P/D614G (100% pseudouridine), Delta S2P, and Delta S6P mutation were utilized for transfection. Notably, the S6P-stabilized spike protein exhibited an elevated expression level compared to S2P in the WT and Delta variants (**Figure 1A**; **Supplementary Figure 2**). The two bands observed in WT 2P are due to the absence of furin cleavage site mutations R682S and R685G, which are present in the Delta 2P and 6P variants. The mRNA encoding the S6P-stabilized SARS-CoV-2 spike protein demonstrated the highest expression and manifested robust binding activity to hACE2 and Clone H4 when formulated with lipid mixture, designated as RV-1730 (**Figures 1B–D**). These findings imply that RV-1730, featuring the S6P-stabilized spike protein, holds considerable promise as a vaccine candidate against SARS-CoV-2, with the potential to induce robust immunogenic responses *in vivo*.

### RV-1730 induces robust humoral responses in mice compared to S2P-based vaccines

**Figure 2:**
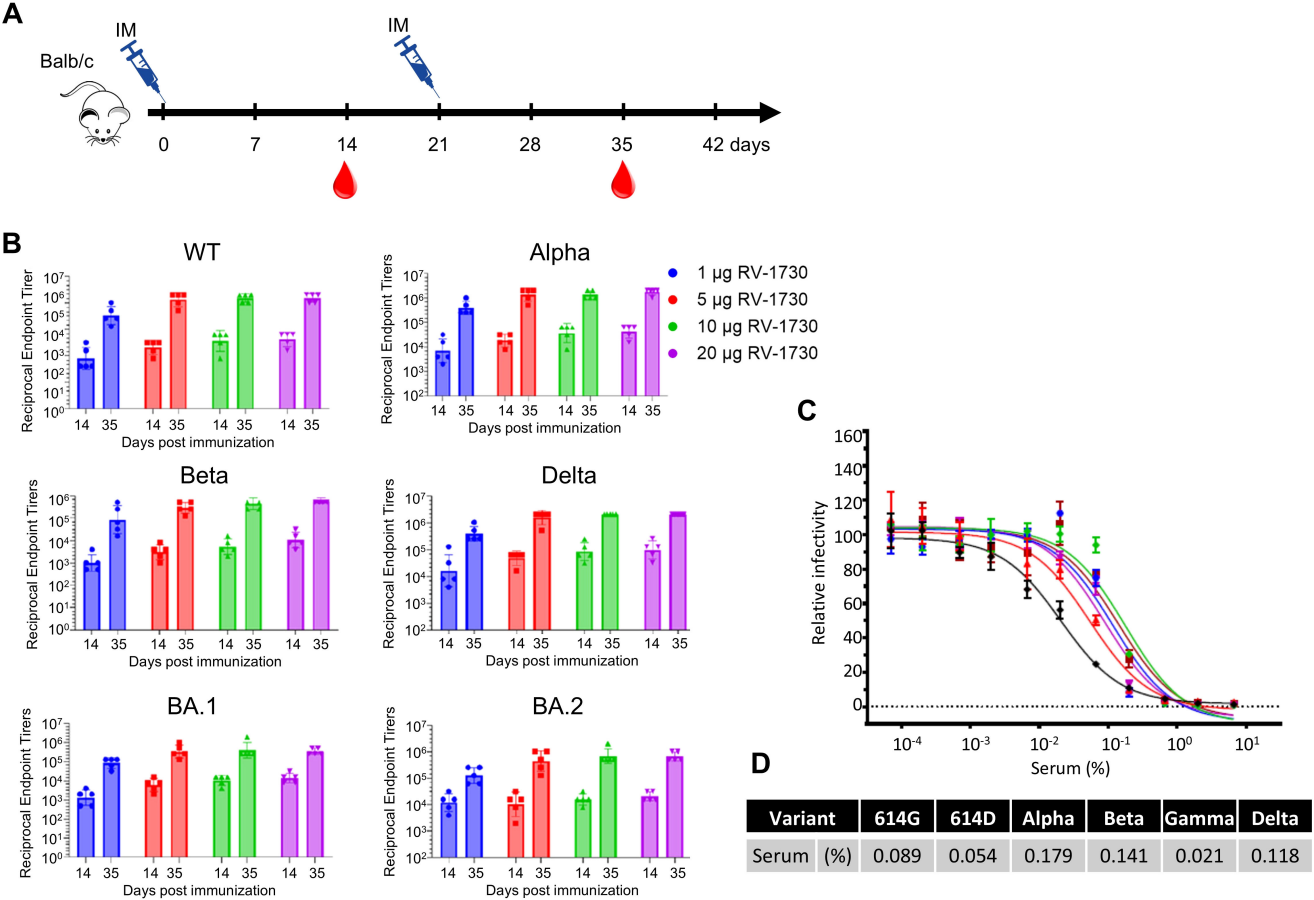
Endpoint IgG titers and neutralizing antibody titers of RV-1730 immunized sera against different variant spike proteins. **(A)** Immunization schedule of animal experiment. Female Balb/c mice were randomly divided into groups of mice (n = 5~8) and vaccinated via intramuscular injection with RV-1730. All mice were immunized twice at 21-day intervals. Blood was taken before each immunization at 14 and 35 days after the first immunization via submandibular bleeding. **(B)** Reciprocal endpoint titers of serum samples. Serum samples at the indicated time were tested in ELISA against S1 protein of WT, Alpha, Beta, Delta, Omicron BA.1, And BA.2. Each symbol represents a serum sample, and the bar is the geometric mean of the group. **(C)** Serum neutralizing titers of the serum in a pseudovirus neutralization assay. Pooled serum samples from the 10 µg group (n=5), collected on day 35, were tested using pseudovirus particles representing various variants. **(D)** The 50% inhibitory dilution titers (IC_50_) were calculated as the percentage of the diluted serum.

We then examined the immunogenicity of the RV-1730 Delta variant with S6P mutations and assessed functional immune responses, such as IgG titer and neutralizing antibody, against various SARS-CoV-2 variant spike proteins, including the WT, Alpha, Beta, Delta variants, and Omicron BA.1 and BA.2 variants. In brief, naive female Balb/c mice aged 6 to 8 weeks were intramuscularly vaccinated with RV-1730 at doses of 1, 5, 10, and 20 μg per mouse on day 0 and day 21 (**Figure 2A**). ELISA was used to evaluate mouse IgG titers against SARS-CoV-2 spike protein fragment S1. RV-1730 induced robust IgG titers against SARS-CoV-2 S1 for the WT, Alpha, Beta, Delta, and Omicron BA.1 and BA.2 variants (**Figure 2B**). The results demonstrated dose-dependent IgG titers across all variants, albeit with slight variations in intensity. To determine if the serum from immunized mice with RV-1730 could inhibit SARS-CoV-2 infection, HEK293T cells were stably transfected with ACE2 and exposed to SARS-CoV-2 pseudotyped particles (PP) from murine leukemia virus (MLV). MLV pseudovirus expressing spike proteins from pcDNA3.1-SARS-CoV-2 vectors was used for spike protein expression. The blood sample from the group exhibiting the highest IgG titer against SARS-CoV-2 spike protein S1 and RBD (e.g., RV-1730 immunized at 10 μg) 35 days post-immunization was utilized in the neutralization assay. In these assays, the serum effectively prevented infection of HEK293-ACE2 cells by very low concentrations of SARS-CoV-2 pseudotyped particles (PP) from different variants (WT, D614G, Alpha, Beta, and Delta) (**Figure 2C**). At 35 days post-immunization, the serum exhibited potent neutralizing activity against all tested variants, with a range of 559 – 4,762 serum IC_50_ in GMT (**Figure 2C**). The Gamma variant was the most sensitive, while the Alpha variant was the most resistant to RV-1730-immunized serum (0.021 and 0.179% of IC_50_, respectively) (**Figure 2D**). RV-1730 demonstrated strong immunogenicity against early SARS-CoV-2 strains in mice.

### RV-1730 induces strong and Th1/Th2-balanced T cell responses in mice

**Figure 3:**
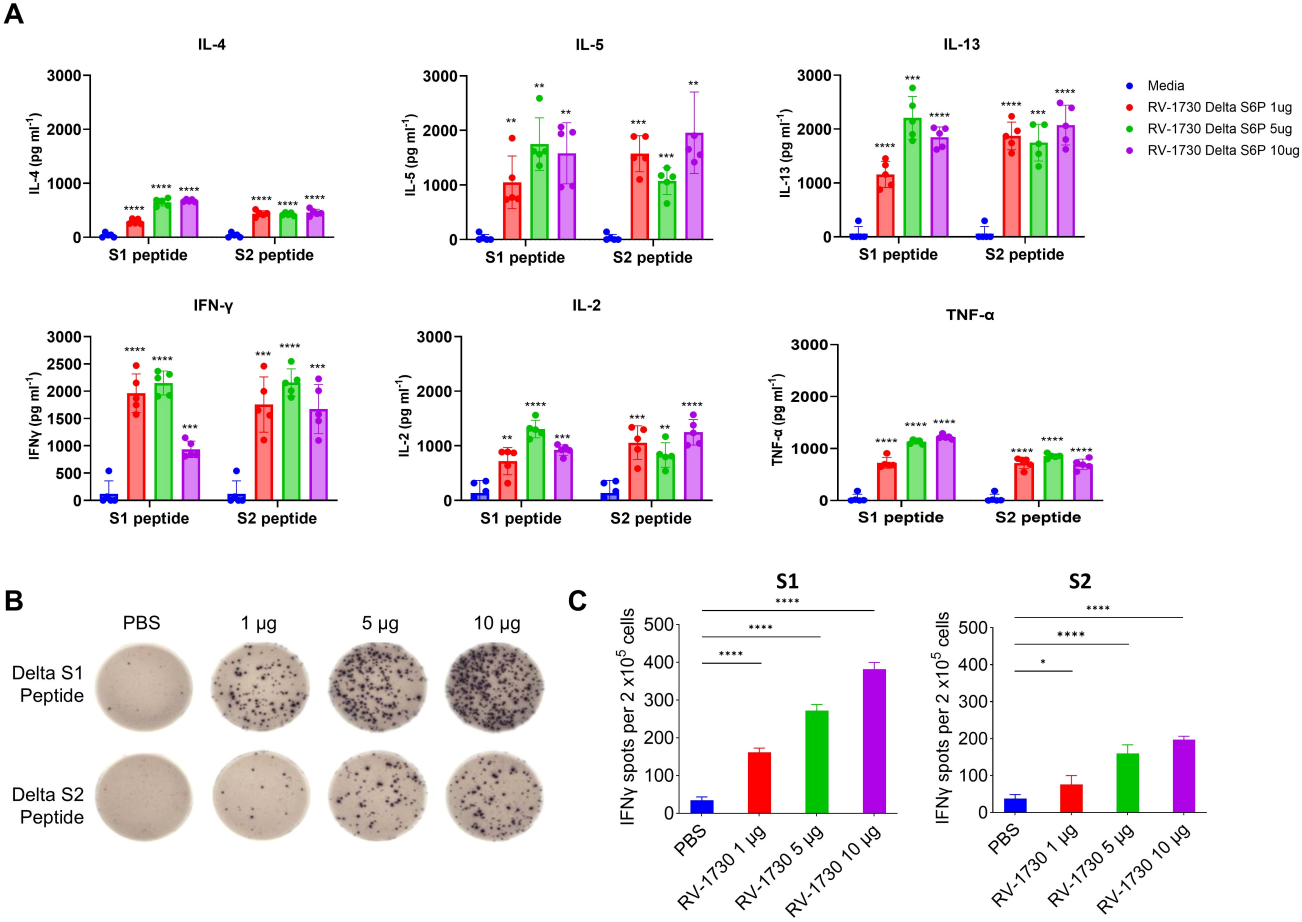
T cell responses in mice immunized with RV-1730. **(A)**. 9 weeks post-boost, splenocytes were isolated from 5 mice per group and re-stimulated with no peptides or pools of overlapping peptides from SARS-CoV-2 S protein. After 72 hours, the culture supernatants were harvested by centrifugation and the secreted Th1-cytokines (IFN-γ, IL-2, TNF-α) and Th2-cytokines (IL-4, IL-5, IL-13) were measured using the bead-based, 11-plex TH1/TH2 mouse ProcartaPlex multiplex immunoassay (Thermo Fisher Scientific). Fluorescence was measured with a Luminex100/200 system and analyzed with ProcartaPlex Analyst 1.0 software (Thermo Fisher Scientific). Below the lower limit of quantification were set to zero. **(B, C)** IFN-γ ELISPOT analysis on splenocytes. Splenocytes (2 x10^5^ cells per 96 well) were plated onto mouse IFN-γ ELISpot plates (Mabtech) and re-stimulated *ex vivo* with pools of overlapping peptides from SARS-CoV-2 Delta Spike for 16 hours. Images were taken **(B)** and quantified by using Cytation7 **(C)**. *P<0.05 and ****P<0.0001 (one-way ANOVA with Dunnett’s test). **p<0.01, ***p<0.001.

We further evaluated T cell immune responses to RV-1730 in mice. Mice were immunized with RV-1730 at doses of 1, 5, and 10 μg per mouse, and their sera were analyzed for antibody isotypes. Across all three concentrations tested, RV-1730 immunogens generated both IgG2a/c and IgG1 subclass S-binding antibodies, indicating a balanced Th1/Th2 response (**Supplementary Figure 3**). This observation was further validated through a multiplex cytokine secretion assay using the Luminex 100/200 system. Upon restimulation with peptide pools (S1 and S2) corresponding to the S protein, splenocytes from RV-1730 immunized mice exhibited a well-balanced secretion of Th1 cytokines (IFN-γ, IL-2, TNF-α) and Th2 cytokines (IL-4, IL-5, IL-13) (**Figure 3A**), demonstrating a dose-dependent response. Quantification of SARSCoV-2 antigen-specific IFNγ-producing T cells by ELISpot revealed a robust induction of IFN-γ+ ELISpots, particularly with the Delta S1 peptide pool, while S2 peptide pools exhibited a comparatively lower frequency (**Figures 3B, C**). Further assessment of cytokine patterns in vaccine-induced memory T cells through intracellular cytokine staining (ICS) confirmed that the IFN-γ+ T cell response, stimulated *ex vivo* with Delta S1/S2 peptide pools, supported the notion that the Delta S1 peptide pool induced activation of both CD4+/IFN-γ+ and CD8+/IFN-γ+ T cells (**Supplementary Figure 4**). These findings collectively indicate that the RV-1730 vaccine can elicit a strong T cell immune response, characterized by T cells capable of producing a balanced Th1/Th2 cytokine profile.

### Immunization of RV-1730 provides complete protection against SARS-CoV-2 ancestral WT strain challenge in mice

**Figure 4:**
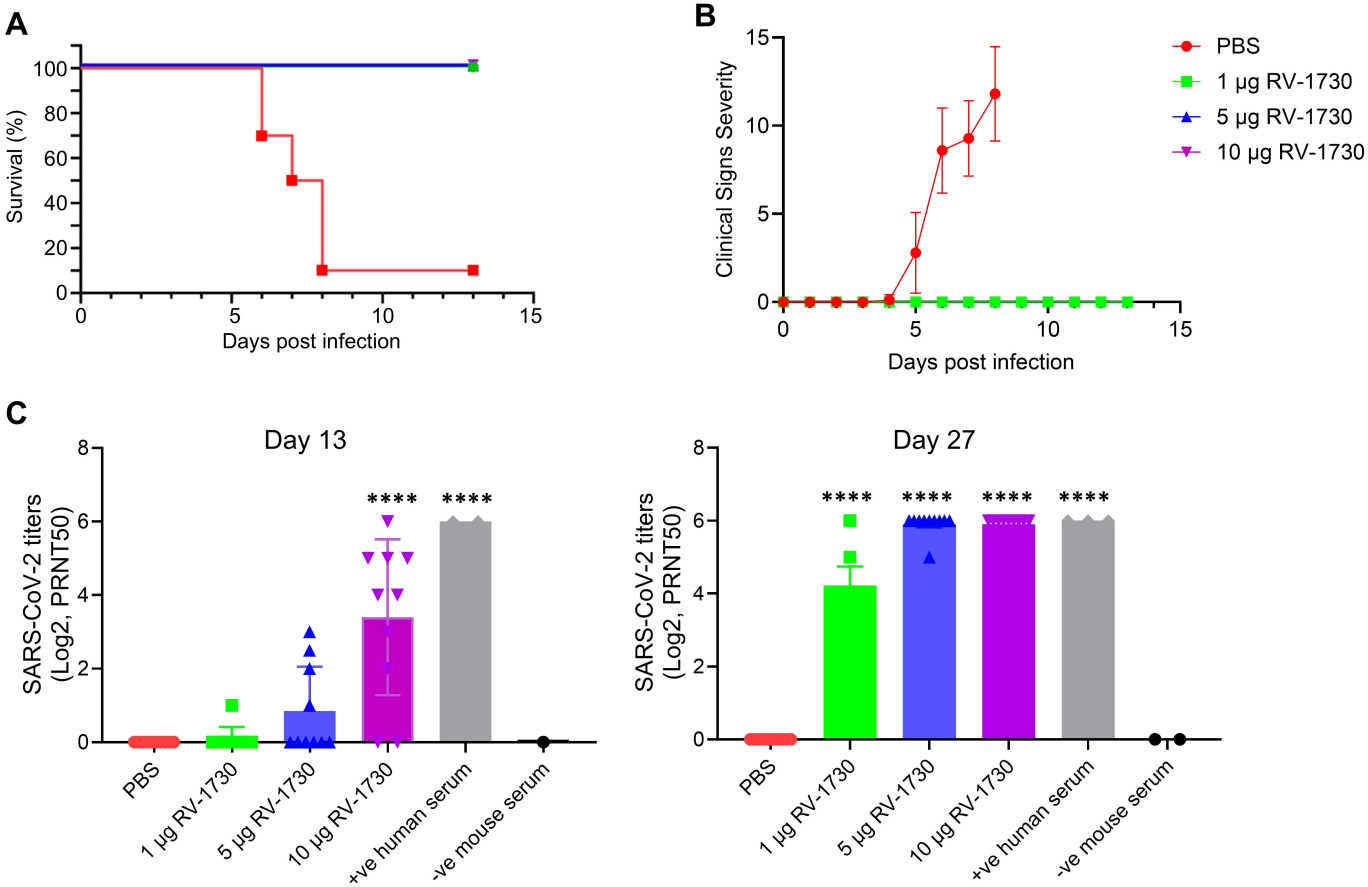
Protective efficacy of RV-1730 against SARS-CoV-2/human/ITA/INMI1/2020 in a K18-hACE2 mouse model of lethal infection. K18-ACE2 mice (n = 10 per group) were immunized with 3 doses of 1, 5, and 10 µg RV-1730 per mouse and challenged with wild type SARS-Cov-2 virus as described in “Methods” section. Mice in Group 1 were administered PBS, Group 2 mice were immunized with mRNA RV-1730 1 µg/animal, Group 3 mice were immunized with mRNA RV-1730 5 µg/animal, Group 4 mice were immunized with mRNA RV-1730 10 µg/animal before viral challenge. **(A)** Kaplan Meier curve of survival rate for mice challenged with SARS-CoV-2/human/ITA/INMI1/2020 virus. **(B)** clinical severity score curve in immunized and non-immunized mice after challenge with SARS-CoV-2 virus. **(C)** Plaque reduction neutralizing test of mouse serum against SARS-CoV-2/human/ITA/INMI1/2020. PRNT_50_ of post-prime immunization mouse serum collected on day 13 and day 27. Symbols and horizontal lines represent individual titers of each sample and mean titers of each group, respectively. Serum titers were expressed as reciprocals Log_2_ dilution. ****P<0.0001 compared to PBS (one-way ANOVA with Dunnett’s test).

In a comprehensive assessment of the RV-1730 vaccine’s efficacy and dose-response, we utilized a mouse model of lethal infection by immunizing human ACE2 knocked-in K18-hACE2 mice with RV-1730. The immunizations were administered via intramuscular injection into the hind leg on study days 0 (prime) and 14 (boost). In this challenge study, we shortened the dosing interval from 21 to 14 days to expedite the study timeline and evaluate immune responses within a shorter timeframe. This adjustment was based on preliminary data indicating that a robust immune response could still be achieved with the condensed schedule. On day 28, the mice were transferred to ABSL-3 containment and intranasally challenged with 5.5 x 10^4^ PFU/mouse SARS-CoV-2/human/ITA/INMI1/2020 (wild type). Throughout the thirteen-day post-challenge period, all animals were diligently monitored for mortality, body weight, body temperature, and clinical signs of distress. Immunization with three different doses of RV-1730 demonstrated complete protection against lethal infection (**Figure 4A**), with no observed clinical distress or loss of body weight in any of the immunized groups (**Figure 4B**; **Supplementary Figures 5**, **6**). In contrast, the non-immunized control group exhibited severe clinical distress, leading to the euthanization of 90% of the mice due to high post-SARSCoV-2 challenge clinical severity scores (>10) (**Figure 4B**). Additional evaluation using a plaque reduction neutralization test (PRNT) against the SARS-CoV-2/human/ITA/INMI1/2020 (WT) virus showed significant titers of neutralizing serum antibodies at both 13 and 27 days post-prime immunization (**Figure 4C**). High-dose mRNA RV-1730 immunization (10 μg/mouse) induced a substantial level of neutralizing serum antibodies against SARS-CoV-2 after the prime immunization on day 0. Lower doses (1 μg/mouse and 5 μg/mouse) resulted in undetectable or low levels of neutralizing antibodies after the prime immunization but boosted immunizations (day 14) increased serum neutralizing antibody titers for all doses, with 5 μg/mouse and 10 μg/mouse doses yielding higher levels compared to the 1 μg/mouse dose. Prime-boost immunization at all three dose levels provided complete protection against lethal SARS-CoV-2 challenge and induced neutralizing serum antibodies. Histopathology analysis of lung tissue specimens indicated substantial reductions in total lung pathology and interstitial pneumonia, suggesting protection from the inflammatory effects of SARS-CoV-2 infection (**Supplementary Figure 7**). Overall, RV-1730 immunization in female K18-hACE2 mice provided robust protection against SARS-CoV-2 WT, inducing neutralizing antibodies and mitigating pathological effects, supporting its potential as an effective vaccine candidate.

### Immunization of RV-1730 provides better protection against SARS-CoV-2 Delta challenge in mice than WT S2P vaccine

**Figure 5:**
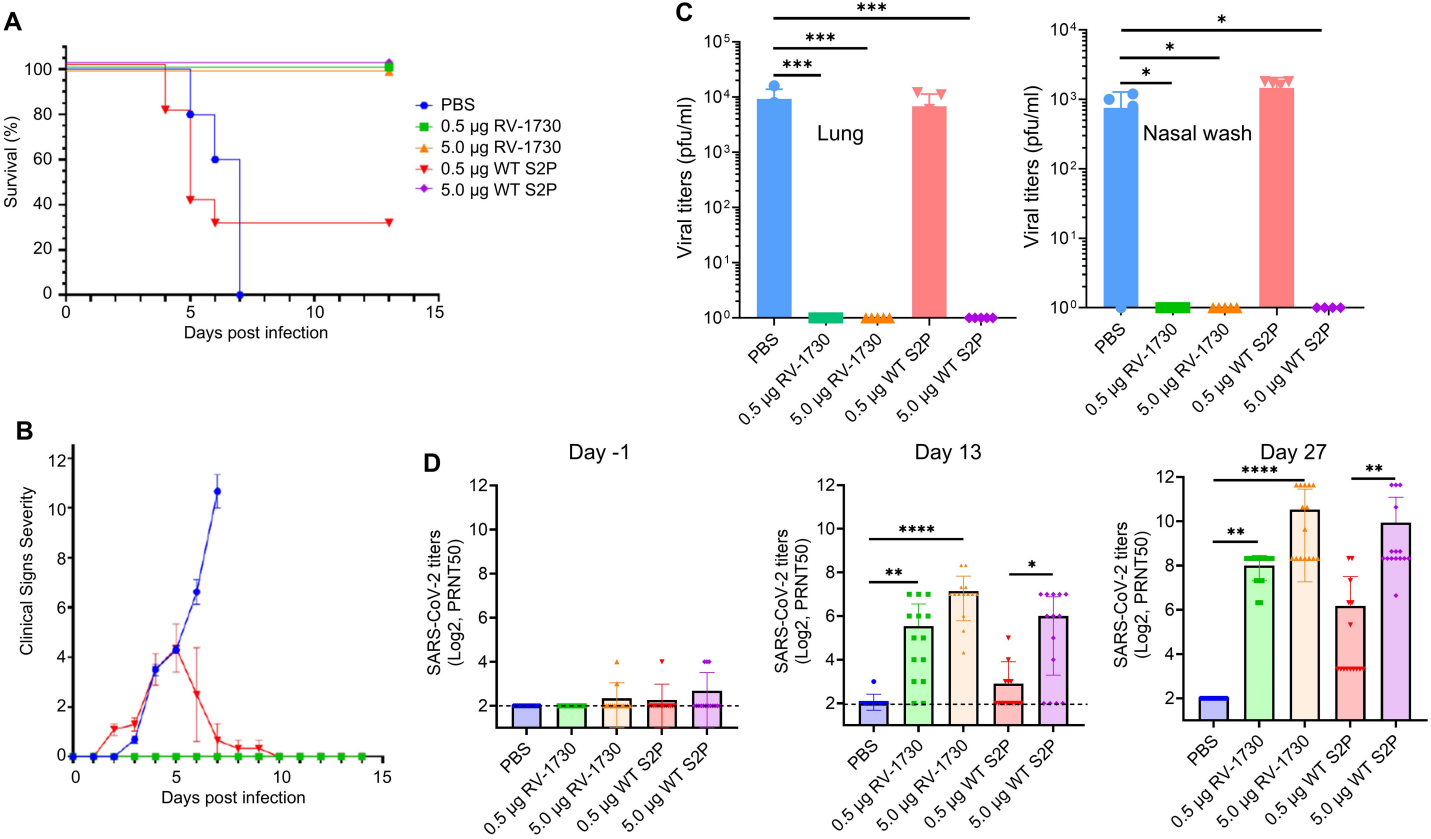
Protective efficacy of RV-1730 against SARS-CoV-2 B.1.1617.2 Delta variant/in a K18-hACE2 mouse model of lethal infection compared with WT S2P. **(A)** Survival rate for mice challenged with SARS-CoV-2 B.1.1617.2 Delta variant (n=10). Group 1 PBS control. Groups 2 and 3 – immunized with 0.5 µg or 5.0 µg RNAImmune mRNA vaccine, respectively. Groups 4 and 5 immunized with 0.5 µg or 5.0 µg WT S2P control mRNA, respectively. Kaplan Meier curve. **(B)** Clinical Severity Score Curve in immunized and non-immunized mice after challenge with SARS-CoV-2 B.1.1617.2 Delta Variant. **(C)** Post-SARS-CoV-2 viral load in lung tissue and nasal washes (n=5). Viral load in lung tissues and nasal washes harvested day 3 post-infection determined by plaque assay (left), viral load in nasal washes collected day 3 post-infection determined by plaque assay (right). Symbols and horizontal lines represent individual titers of each mouse and mean of each group, respectively. Any value less than 1 x 10^1^ PFU/ml in (left) and any value less than 1 x 10^2^ PFU/ml in (right) was considered as 0. **(D)** Mouse serum titration against SARS-CoV-2/Delta (n=15). PRNT_50_ of mouse serum collected on day -1 (left), day 13 (middle), and day 27 (right)post-boost immunization. *P<0.05, **P<0.01, ***P<0.001 and ****P<0.0001 (one-way ANOVA with Dunnett’s test).

Subsequently, we sought to compare the efficacy of S6P RV-1730 with that of the wild type S2P vaccine (WT S2P) equivalent to mRNA-1273. The protective potential of RV-1730 was assessed in female K18-hACE2 mice, with two different doses (0.5 μg/mouse and 5 μg/mouse) administered. Mice underwent prime immunization on day 0 and a booster shot on day 14, receiving either 0.5 μg/mouse or 5.0 μg/mouse of RV-1730 or WT S2P that has the same sequence as the mRNA-1273. On day 28, intranasal challenges were conducted with 1.5 x 10^4^ PFU/mouse SARS-CoV-2/Delta variant. Prime-boost immunization with either 0.5 μg/mouse or 5.0 μg/mouse of RV-1730 and 5.0 μg/mouse of WT S2P vaccine conferred complete protection against SARSCoV-2 Delta (**Figure 5A**), preventing mortality, clinical distress, and weight loss (**Figure 5A**; **Supplementary Figure 8**). In contrast, the non-immunized control group exhibited distress, body weight loss in all mice, and necessitated euthanization due to the severity of post-SARS-CoV-2 Delta challenge clinical distress. Post-challenge viral loads were detected in the non-immunized group and the group immunized with 0.5 μg/mouse WT S2P but not in mice immunized with either dose of RV-1730 or the 5.0 μg/mouse dose of WT S2P in lung and nose (**Figure 5B**). Immunization with either dose of RV-1730 or 5.0 μg/mouse of WT S2P resulted in a dose-dependent increase in neutralizing titers against SARS-CoV-2/Delta in serum collected on days 13 and 27 (**Figure 5C**). In contrast, prime-immunization and boosting with PBS control or 0.5 μg/mouse of WT S2P vaccine resulted in undetectable or lower levels of neutralizing activity in both day 13 and day 27 sera (**Figure 5D**). The higher neutralizing activity in day 27 sera correlated with the 14-day survival outcome of the challenged mice (**Figure 5A**). Histopathology analysis of lung tissue specimens revealed an increased total lung pathological score, interstitial pneumonia, and perivascular cuffs in the PBS control group and the group immunized with 0.5 μg/mouse WT S2P vaccine compared to the groups immunized with RV-1730 vaccine or 5.0 μg/mouse WT S2P vaccine, both in the survival arm of the study and in the lungs of mice (n=4~5) euthanized on day 3 post-challenge (**Supplementary Figure 9**). All together, these findings indicate that S6P RV-1730 immunization provides superior protection against SARS-CoV-2 Delta challenge in mice compared to the WT S2P vaccine.

### Heterologous vaccination with RV-1730 provides benefits after primary vaccination with BNT162b2 and mRNA-1273

**Figure 6:**
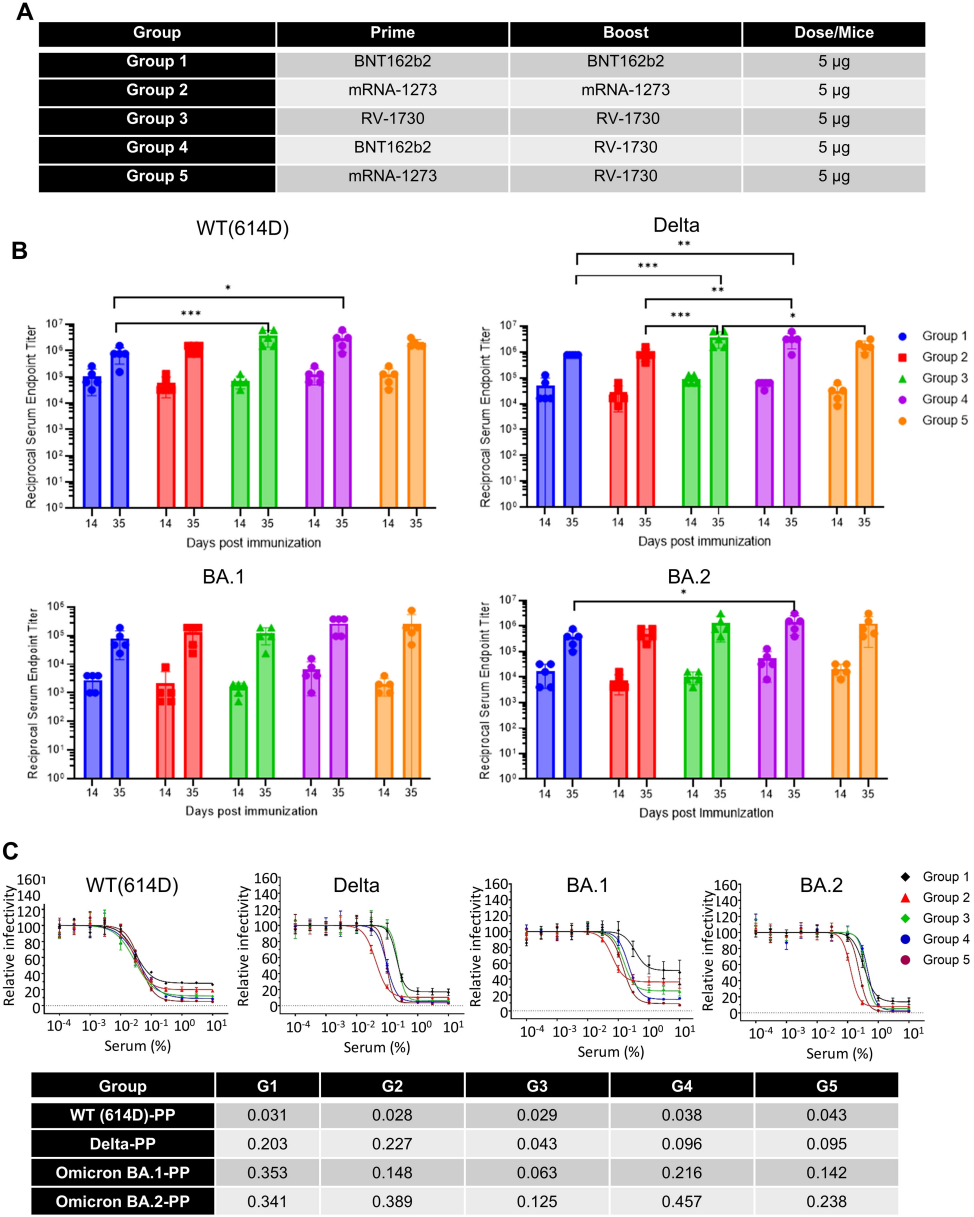
IgG and neutralization titers of homologous and heterologous SARS-CoV-2 spike mRNA-LNP immunized sera. **(A)** Homologous and heterologous immunization with different vaccines. **(B)** Endpoint IgG titers were determined for homologous and heterologous SARS-CoV-2 spike mRNA-LNP immunized sera against distinct variant spike RBD proteins. Mice (n=5) received prime immunization with SARS-CoV-2 spike mRNA-LNP (BNT162b2, mRNA-1273, and RV1730, 5 μg/mouse) and boost immunization either with the same vaccine (homologous) or a combination of vaccines (heterologous) three weeks after the prime immunization. Serum samples were collected on day 14 and 35 days to assess antibody levels specific to SARS-CoV-2 WT, Delta, Omicron BA.1 and BA.2 RBD proteins through ELISA. **(C)** The neutralization antibody titer against SARS-CoV-2 pseudovirus particles was analyzed, with the x-axis representing serum concentration and the y-axis indicating relative pseudoparticle infectivity. Different pseudovirus particles (PP), including 614D-PP, Delta-PP, Omicron BA.1-PP, and BA.2-PP, were used in the assay. The bottom table presents the IC_50_ (serum %) of heterologous vaccination sera in the pseudovirus neutralizing assay. *P<0.05, **P<0.01, and ***P<0.001 (two-way ANOVA with Tukey’s test).

Next, we aimed to assess the immunogenicity of sera resulting from both homologous and heterologous vaccinations and to evaluate functional neutralizing responses using a pseudovirus assay against various SARS-CoV-2 variants. Female Balb/c mice received intramuscular vaccinations with homologous (Group 1-3) and heterologous (Group 4 and 5) doses of BNT162b2, mRNA-1273, and RV-1730 at 5 µg per mouse, administered twice at 21-day intervals (**Figure 6A**; **Supplementary Figure 10**). Reciprocal serum endpoint IgG titers were determined through ELISA, employing a 2-fold serial dilution of the immunized sera. Prime-boost vaccination with homologous doses of BNT162b2, mRNA-1273, and RV-1730 generated robust IgG titers against the WT, Delta, BA.1, and BA,2 receptor-binding domain (RBD) of SARS-CoV-2 S (**Figure 6B**). Notably, homologous immunization with RV-1730 (group3) at day 35 post-immunization significantly increased IgG titers compared to either homologous immunization with BNT162b2 or mRNA-1273 against WT, Delta RBD (P < 0.001) (**Figure 6A**). In particular, heterologous immunization with RV-1730 in group 4 and 5 showed slightly an increased IgG titer against Delta as well as BA.2 RBD compared to those of homologus immunization of BNT162b2 and mRNA-1273. In pseudovirus neutralizing assays, serum samples derived from the Group 3, homologous immunization of RV-1730 effectively blocked infection from different variants (WT D614G, Delta, Omicron BA.1 and BA.2) to HEK293-ACE2 cells at very low concentrations compared to those of other groups derived from either homologus(Group1 and 2) or heterologous(Group 4 and 5). At 35 days post-immunization, the serum displayed strong neutralizing activity against all tested variants, with a range of 0.02-0.18% serum of IC_50_ (**Figure 6C**). These findings indicate that RV-1730 induces a strong and broadly neutralizing immune response, effectively inhibiting multiple SARS-CoV-2 variants—including WT D614G, Delta, and Omicron (BA.1 and BA.2)—even at low serum concentrations.

### The booster vaccination of commercial primary vaccines with RV-1730 elicits robust immune responses

**Figure 7:**
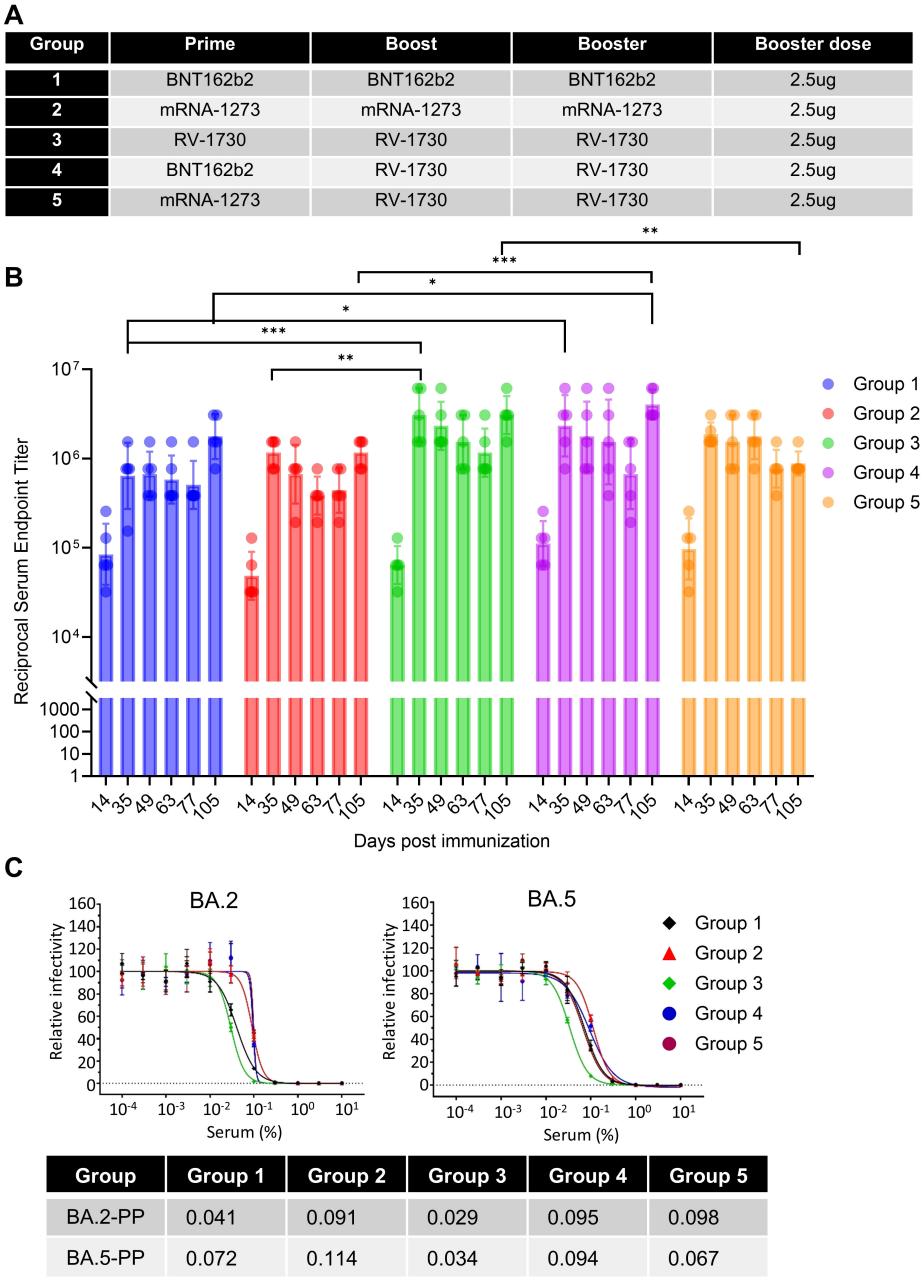
Serological evaluation of the booster vaccination of commercial primary vaccines with RV-1730. **(A)** Booster immunization using different vaccines in homologous and heterologous vaccinated mice. **(B)** Endpoint IgG titers against wild-type spike S1 proteins were measured on day 105 in sera from booster-immunized mice. Mice (n=5) were initially immunized with SARS-CoV-2 spike mRNA-LNP vaccines (BNT162b2, mRNA-1273, or RV1730) and received booster doses either with the same vaccine (homologous, Groups 1-3) or a combination of different vaccines (heterologous, Group 4) on day 0 (5 μg/mouse), day 21 (5 μg/mouse), and day 91 (2.5 μg/mouse), as detailed in **Supplementary Figure 10**. Serum samples collected on day 105 were analyzed for SARS-CoV-2 RBD-specific antibody levels using ELISA. *P<0.05, **P<0.01, and ***P<0.001 (two-way ANOVA with Tukey’s test). **(C)** The neutralizing antibody titer against SARS-CoV-2 pseudovirus particles is shown. The X-axis represents compound concentration, while the Y-axis indicates relative pseudovirus particle (PP) infectivity. The neutralizing activity of booster (third dose) vaccinated sera against BA.2 and BA.5-PP was assessed on day 105. The table below displays the IC_50_ values for heterologous vaccination sera in the pseudovirus neutralization assay.

**Figure 8:**
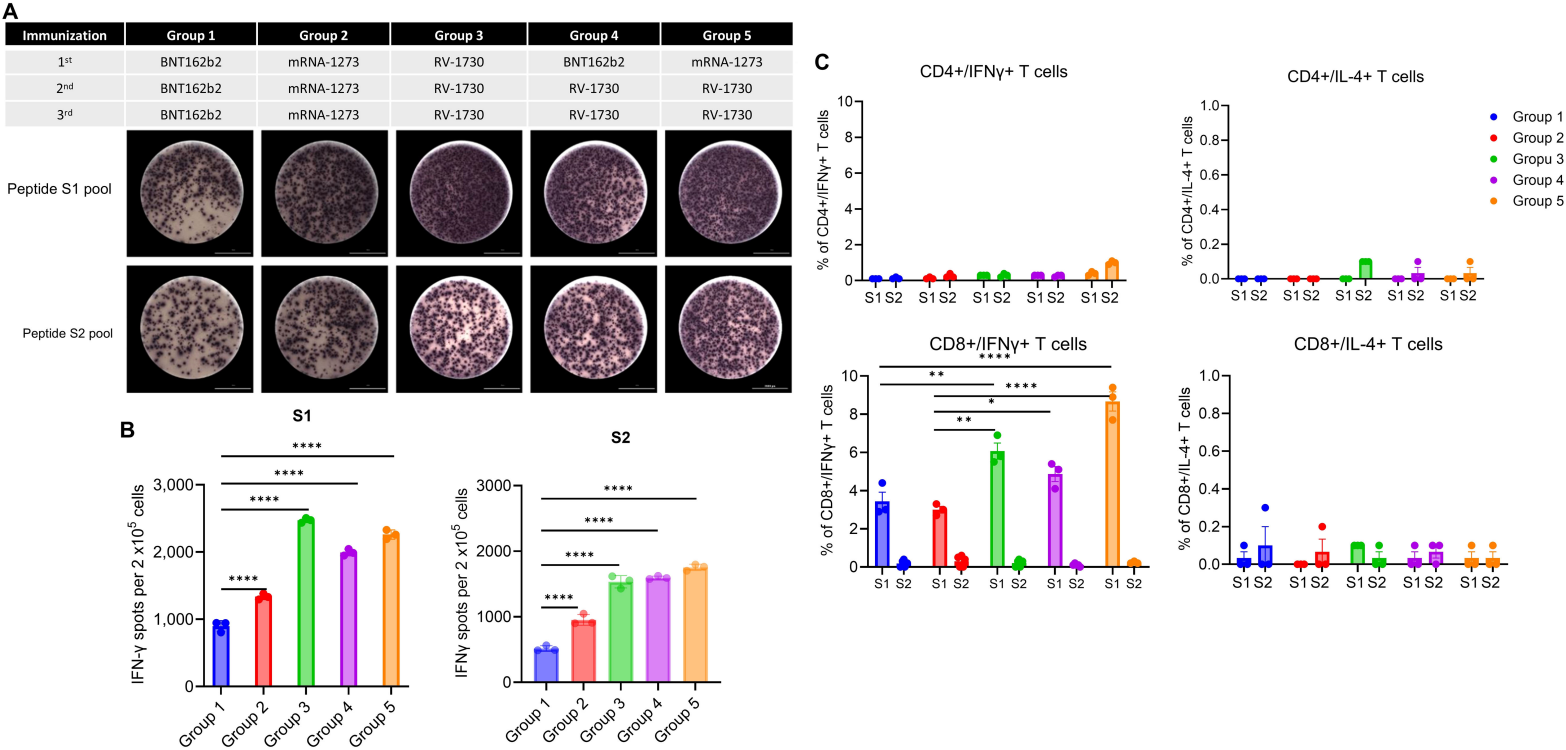
T-cell responses in mice vaccinated with RV-1730 booster administration. **(A)** IFN-γ ELISPOT analysis was conducted on splenocytes. Two weeks after the administration of the third booster shot, splenocytes were harvested from five mice per group. These splenocytes (2 x 10^4^ cells per 96-well plate) were then seeded onto mouse IFN-γ ELISpot plates (Mabtech) and ex vivo re-stimulated with pools of overlapping peptides derived from the SARS-CoV-2 Delta Spike for a duration of 16 hours. Subsequently, images were captured and quantified using Cytation7. Presented is a representative image of the ELISpot. **(B)** The quantitative analysis of the ELISpot data revealed statistical significance. **(C)** CD4/CD8 T cell analysis. The splenocytes were isolated and activated following the procedures outlined in the ELISPOT assays. The upper row illustrates the gating steps for CD3+ CD4+ and CD3+ CD8+ cell populations. IFN-γ+ and IL-4+ within the CD4 and CD8 subpopulations were analyzed by a flow cytometer. The percentage of CD4/IFN-γ, CD4/IL-4, CD8/IFN-γ, and CD8/IL-4 cells are presented. *P<0.05, **P<0.01, and ****P<0.0001 (one-way ANOVA with Dunnett’s test).

Homologous and heterologous vaccinated mice received a booster shot with BNT162b2, mRNA-1273, and RV-1730 at day 72 post 2^nd^ immunization. Blood samples were collected before each immunization at 14, 35, 49, 63, 77, and 105 days after the initial immunization (**Figure 7A**; **Supplementary Figure 10**). In booster immunization, both homologous and heterologous immunization induced strong IgG endpoint titers against SARS-CoV-2 S S1 of WT. Although heterologous booster immunization with BNT162b2 and RV-1730(Group 4) at day 105 significantly increased IgG titers against WT S1 compared to homologous immunization (Group 1, P < 0.05and Group 2, P <0.001) (**Figure 7B**). Only primary and booster vaccination with RV-1730 demonstrated a potent neutralizing antibody response against Omicron BA.2 and BA.5 (**Figure 7C**). RV-1730 exhibited approximately a 10-fold increase in neutralizing activity against Omicron BA.1 and BA.5 compared to sera before booster immunization. Additionally, homogenous immunization with RV-1730 as a booster showed a strong induction of IFN-γ+ CD8 T cell response by Delta S1 peptide pool compared to homogenous boosters with BNT162b2 and mRNA-1273 (**Figures 8A, B**). Furthermore, heterogenous boosting with RV-1730 following primary vaccination with BNT162b2 and mRNA-1273 enhanced the induction of IFN-γ+ CD8 T cell response by Delta S1 peptide pool (**Figure 8C**). Given that RV-1730 monovalent booster immunization exhibited comparable neutralizing activity to BA.5 primary vaccination, the variant-modified booster vaccination with RV-1730 can provide significantly higher neutralization titers against a diversity of current (e.g., BA.5) and historic SARS-CoV-2 variants compared to ancestral-based boosters. The enhanced responsiveness observed with RV-1730 may be influenced by the use of stimulating peptides that are homologous to the vaccine’s Delta variant sequence. In contrast, BNT162b2 and mRNA-1273, which are based on the ancestral strain, may show reduced responsiveness in this assay due to the heterologous nature of the peptides used (**Figure 8C**). In conclusion, booster immunization with RV-1730 not only elicited robust IgG responses against diverse SARS-CoV-2 variants but also demonstrated superior T cell activation, highlighting its potential as an effective and comprehensive vaccination strategy.

### RV-1731, combining Delta and BA.1 spike with S6P, reflects the neutralization effectiveness akin to booster doses of the commercial bivalent vaccine

**Figure 9:**
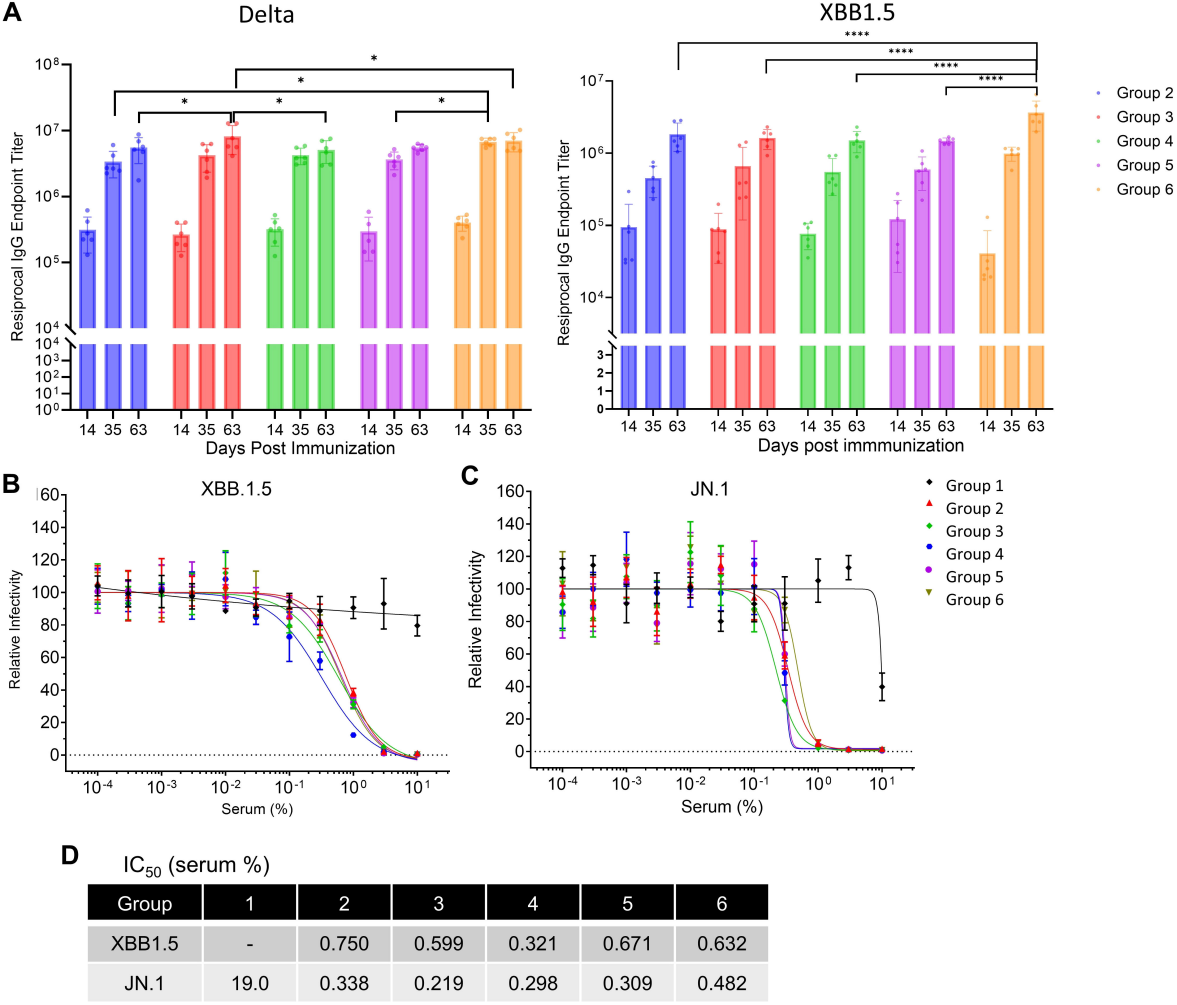
Inhibitory effect of RV1731’s mouse serum SARS-CoV-2 XBB.1.5 and JN.1 PPs infection. **(A)** Endpoint IgG titers of sera from booster immunizations with SARS-CoV-2 spike mRNA-LNP vaccines targeting Delta and XBB1.5 spike RBD proteins. The sera were collected from the animal study shown in **Supplementary Figure 11**. *P<0.05 and ****P<0.0001 (two-way ANOVA with Tukey’s test). **(B, C)** The graphs display the neutralizing activity of booster-immunized vaccines against two SARS-CoV-2 variants: XBB1.5 **(B)** and JN.1 **(C)**. Sera from 6 mice per group were collected and subjected to a pseudovirus neutralization assay. **(D)** The IC_50_ (serum %) was determined using GraphPad software by analyzing the curves for each serum concentration.

Despite multiple doses of the original WT vaccine, the neutralizing antibody efficacy against new variants significantly decreased, prompting two major vaccine companies to develop a bivalent booster vaccine targeting WT and Omicron BA.5 during the pandemic. In response, we developed a bivalent vaccine incorporating Delta and Omicron BA.1 and conducted *in vivo* immunogenicity studies in mice to compare its efficacy with Pfizer-BioNTech and Moderna’s bivalent vaccine (Original and Omicron BA.4/BA.5). This bivalent formulation is designed to provide broader protection against newer variants, which are increasingly resistant to earlier vaccines. The subsequent results focus on the immunogenicity and neutralizing efficacy of RV-1731 Both homologous and heterologous bivalent booster immunizations (**Supplementary Figure 12**) elicited robust IgG endpoint titers against the RBDs of SARS-CoV-2 Delta, and XBB1.5 variants (**Figure 9A**). Notably, at day 63 post-booster, sera from individuals immunized with RV-1730 and RV-1731 (group 6) showed significantly higher IgG titers against XBB1.5 RBD compared to all other immunization groups (**Supplementary Figure 13**). Neutralizing activity of the sera was assessed using pseudovirus assays against the XBB1.5 and JN.1 variants of SARS-CoV-2. Furthermore, immunization with RV-1730 and RV-1731 (group 6) resulted in a higher IgG titer against Delta RBD compared to group 4 (comprising mRNA-1273 monovalent and mRNA-1273 Original and Omicron BA.4/BA.5 bivalent vaccines). Additionally, booster immunization with RV-1731 demonstrated a robust induction of IFN-γ+ T cell responses against Delta S1 peptide pools, comparable to commercially available commercial bivalent boosters (**Supplementary Figure 14**). In pseudovirus neutralization assays, the RV-1731 booster immunized groups exhibited comparable potency of neutralization to the commercial bivalent vaccines (**Figures 9B, C**). Crucially, administering a booster dose of RV-1731 after the initial vaccination with mRNA-1273, BNT162b2, or RV-1730 monovalent vaccines effectively neutralized the SARS-CoV-2 JN.1 pseudovirus, a variant of concern as of late 2023, comparable to the neutralization achieved with booster immunization using the commercial bivalent vaccines (**Figures 9B–D**). While existing vaccines retain their ability to elicit neutralizing antibody responses relevant to prevailing antigenic strains, the development of vaccines based on the BA.1 and Delta variants with S6P mutations holds promise for enhancing the protective efficacy of vaccine-induced immune responses.

## Discussion

Amidst the continuous emergence of new COVID-19 variants, there’s a growing urgency to develop vaccines that can effectively combat a broad spectrum of strains. This study explores the potential of RV1730 monovalent and RV-1731 bivalent, an LNP formulation containing mRNA encoding the S protein of the SARS-CoV-2 Delta and Omicron BA.1 variant with S6P mutations, to meet this critical need.

The S6P mutation within the COVID-19 spike protein is considered advantageous over the S2P mutation for several reasons: Firstly, it demonstrates increased immunogenicity, meaning it can trigger a stronger immune response, crucial for training the immune system to recognize and combat the virus effectively. Additionally, studies suggest it offers enhanced protection against diverse strains of SARS-CoV-2 by inducing a more potent neutralizing antibody response. Furthermore, the S6P mutation contributes to stabilizing the prefusion conformation of the spike protein, which is vital for preserving the protein’s structure that the immune system relies on for recognition. Vaccines incorporating the S6P mutation have also been associated with more durable neutralizing antibody responses, potentially leading to longer lasting protection and reducing the necessity for frequent booster doses.

Moreover, the S6P mutation allows for the formation of self-assembling nanoparticles when combined with HBsAg, which have demonstrated efficacy in eliciting potent and long-lasting neutralizing antibody responses, surpassing non-nanoparticle forms of stabilized spike (29). Consequently, the inclusion of the S6P mutation in COVID-19 spike vaccines offers potential benefits in terms of immunogenicity, protection, stability, and durability of the immune response, making it a promising candidate for next-generation vaccines (21). While the S6P mutation in the spike protein has been previously reported to enhance the stability and immunogenicity of mRNA vaccines (29), our study advances this by evaluating the specific application of S6P within the Delta variant and in a bivalent formulation targeting both Delta and BA.1. Our findings highlight an even broader neutralizing efficacy across emerging variants, including XBB1.5, thus potentially offering more robust and long-lasting immunity.

Our findings also indicate that RV-1730 and RV-1731 induces potent humoral responses in mice, evidenced by elevated IgG titers against various SARS-CoV-2 variant spike proteins and strong neutralizing antibody activity. Additionally, RV-1730 triggers robust Th1/Th2 T cell responses, as demonstrated by cytokine production and IFN-γ+ T cell generation upon SARS-CoV-2 peptide stimulation (30). While COVID-19 mRNA vaccines typically induce Th1-biased cellular responses (31, 32), our study observed a mixed Th1/Th2 response, possibly due to the unique lipid nanoparticle (LNP) formulation in RV-1730 and RV-1731, which may have modulated the immune response. LNPs have been shown to activate both Th1 and Th2 pathways depending on their composition and immune cell processing (5, 33). Additionally, mutations such as S6P, along with R682S and R685G, may influence antigen processing, leading to broader immune activation (34). The dose and frequency of immunizations may also play a role, as higher doses or repeated booster shots have been reported to activate both Th1 and Th2 pathways (35, 36). While a Th1-biased response is typically preferred for viral clearance, the mixed Th1/Th2 response in our study could offer broader immune protection and mitigate potential risks associated with excessive Th1 polarization (37, 38). Further research is needed to fully understand the mechanisms driving this balanced response and its implications for future vaccine development.

The S6P-modified RV-1730 vaccine offers significantly stronger protection than S2P-based commercial vaccines like mRNA-1273, providing complete defense against SARS-CoV-2 challenges in preclinical models, particularly against the Delta variant. Unlike S2P vaccines, RV-1730 induces more robust and durable immune responses, marked by high levels of neutralizing antibodies and balanced T cell activation across a range of variants, including Delta and Omicron. Additionally, as a booster following primary vaccinations with commercial vaccines (e.g., BNT162b2 or mRNA-1273), RV-1730 further enhances neutralizing activity against multiple variants, underscoring its potential as a more comprehensive and effective vaccination strategy.

However, while RV-1730 shows clear advantages at lower doses, its efficacy at the 5 µg dose appears comparable to that of WT S2P, as indicated by similar neutralizing antibody titers and histopathological findings. This underscores the nuanced nature of immune protection, especially in heterologous challenges, where factors beyond antibody levels—such as T cell responses—may significantly influence overall efficacy.

In our heterologous booster study, the observed cross-protection against Omicron, even after prior immunization with ancestral and Delta strains, warrants further explanation, given that Delta is genetically closer to the ancestral strain. Specifically, Delta exhibits only 7-10 amino acid changes relative to the ancestral strain, while Omicron carries a substantially higher mutation load, with 25-30 changes, particularly within the spike protein’s receptor-binding domain (RBD). Despite this pronounced genetic divergence, our results indicate that the RV-1730 heterologous booster may enhance immune breadth. This effect is likely driven by the S6P stabilization mutation, which improves the presentation of conserved epitopes, thereby broadening antibody responses to include partial recognition of Omicron’s highly mutated RBD. Furthermore, the robust T cell responses elicited by RV-1730 may provide cross-variant protection by focusing on conserved viral regions that are less prone to mutations. These mechanisms collectively help explain the cross-protection observed against Omicron, despite its substantial antigenic distance from both the ancestral and Delta strains.

While RV-1730 demonstrated significant efficacy against the Delta variant, RV-1731 was developed to expand the protective capacity to other major variants, including Omicron BA.1 and XBB1.5. This bivalent approach highlights the importance of versatile vaccines that can adapt to the rapidly evolving viral landscape. From the results, RV-1731 has demonstrated potent immune responses and has proven to be effective in neutralizing the virus, particularly the XBB1.5 and JN.1 variants. Compared to booster doses of the commercial bivalent vaccines, RV-1731 exhibited comparable or superior neutralization abilities, notably against the XBB1.5 variant. Additionally, RV-1731 prompted a strong T cell response, essential for sustained immunity and fighting the virus post-infection. The creation of RV-1731 could significantly improve the efficacy of vaccine-induced immunity, particularly in response to new and emerging variants. As a continuation of RV-1730’s potential, RV-1731 emerges as a strong candidate for enhancing immunity against SARS-CoV-2, with the potential to surpass existing vaccine options. This progress is supported by recent technological advances, such as the ESCRT (Endosomal Sorting Complex Required for Transport) system, which has been pivotal in deepening our understanding of virus-host interactions and improving vaccine delivery mechanisms. ESCRT plays a crucial role in mRNA vaccine packaging, intracellular trafficking, and enhancing antigen presentation—factors critical for optimizing vaccine performance (39). Incorporating such innovations could further enhance the efficacy of next-generation vaccines. Our research highlights the urgent need for developing comprehensive COVID-19 vaccines that can adapt to the evolving variant landscape while ensuring safety and effectiveness in pandemic management.

### Limitations

We acknowledge that this study primarily focused on assessing short-term immune responses; thus, further longitudinal studies are required to evaluate the durability of immunity induced by RV-1730 and RV-1731. Additionally, the preclinical model employed may not fully reflect the complexity of human immune responses, particularly with respect to cross-variant protection and T cell activation. Our research concentrated on specific variants, and while the findings indicate broad potential, further investigation is necessary to confirm efficacy against newly emerging variants. Lastly, the immune response evaluation was largely centered on antibody and T cell responses, without examining other critical components, such as memory B cells. This highlights the need for a more comprehensive immunological assessment in future studies to fully understand the vaccines’ protective capabilities.

## Conclusion

The incorporation of the S6P mutation in the Delta and BA.1 variant spike proteins significantly enhances the efficacy of mRNA-based COVID-19 vaccines, as demonstrated by the robust immune responses and protection observed in preclinical models. RV-1730 and RV-1731 represent promising candidates for next-generation vaccines capable of providing broad and durable immunity against SARS-CoV-2, including emerging variants. Further clinical evaluation is warranted to determine the full potential of these vaccines in combating the ongoing COVID-19 pandemic.

## Data Availability

The datasets used and/or analyzed during the current study are available from the corresponding author on reasonable request. Requests to access the datasets should be directed to DS, dong.shen@rnaimmune.com.
