# Supplementary material for: S6P mutation in Delta and Omicron variant spike protein significantly enhances the efficacy of mRNA COVID-19 vaccines
Source: Front Immunol. 2025 Jan 3;15:1495561. doi: 10.3389/fimmu.2024.1495561 (PMC11739128; doi:10.3389/fimmu.2024.1495561)
Supplement: Supplementary file 1 [file DataSheet1.pdf]

Supplementary Material

S6P Mutation in Delta and Omicron Variant Spike Protein Significantly Enhances the Efficacy of mRNA COVID-19 Vaccines

Yong-Sik Bong[1,3], David Brown[1,3], Ezra Chung[1,3], Neeti Ananthaswamy[1], Renxiang Chen[1,2], Evan Lewoczko[1], William Sabbers[1], Athéna C. Patterson-Orazem[1], Zachary Dorsey[1], Yiqing Zou[2], Xue Yu[2], Jiening Liang[2], Jiaxi He[2], Steven Long[1,*] and Dong Shen[1,*]

[1] RNAimmune, Inc. Germantown, MD, USA

[2] Guangzhou RNAimmune, Ltd. Guangzhou, China

[3] These authors contributed equally to this work.

*** Correspondence:**
Steven Long (stevenlong@rnaimmune.com) and Dong Shen (dong.shen@rnaimmune.com)

Structural overview of spike protein variants

Methods

Sequences corresponding to the extracellular domains of each spike protein variant were modeled in AlphaFold 2 (1) and resulting structural models were assessed in PyMol 2.1 (2). Pre-fusion conformation and model reasonableness were verified using validated experimental pre-fusion spike protein structures accessioned within and validated by the Protein-Data Bank (RCSB.org) (3).

References

1. Jumper, J., Evans, R., Pritzel, A. et al., Highly accurate protein structure prediction with AlphaFold. Nature (2021) 596:583–589. https://doi.org/10.1038/s41586-021-03819-2
2. Schrödinger, L. L. C. (2020). "The PyMOL Molecular Graphics System, Version 2.1." Schrödinger, LLC.
3. H.M. Berman, J. Westbrook, Z. Feng, G. Gilliland, T.N. Bhat, H. Weissig, I.N. Shindyalov, P.E. Bourne, The Protein Data Bank. Nucleic Acids Research (2000) 28: 235-242. https://doi.org/10.1093/nar/28.1.235.

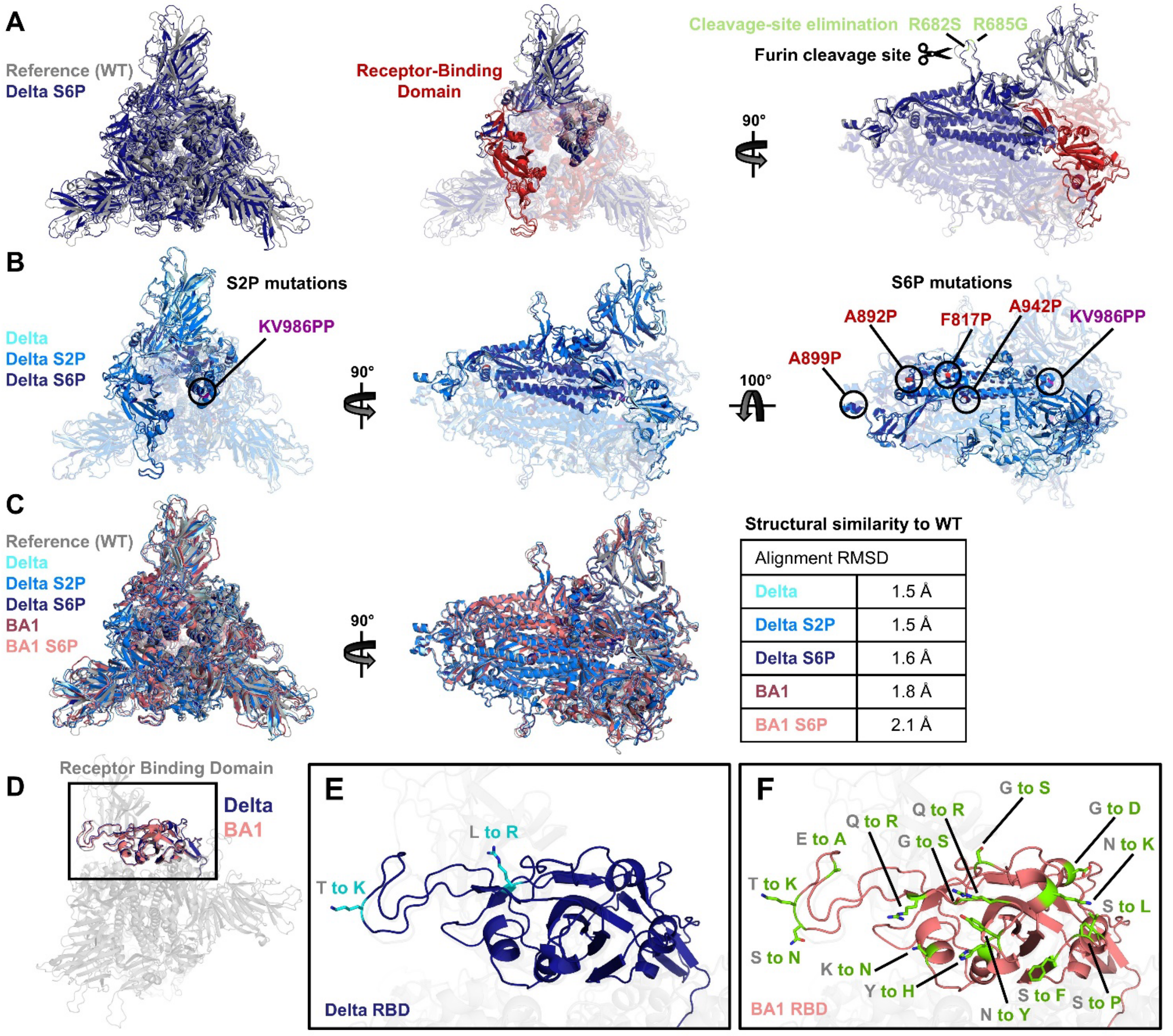

Supplementary Figure 1. Structural overview of spike protein variants. A. Comparison of wild-type (gray) and delta S6P (dark blue) models highlighting the receptor binding domain (red) and mutations eliminating the furin-like cleavage site (green). **B.** Comparison of delta (light blue), S2P (blue) and S6P (dark blue) models highlighting S2P and S6P mutations. **C**. Structural comparison of wild-type, Delta, BA.1 and their stabilizing variants suggests strong structural similarity to wild-type, with RMSD less than 2.5 Å. **D**. A closer look at a receptor-binding domain highlighting mutations compared to wild-type in **E (**Delta) and **F** (BA.1) spike proteins.

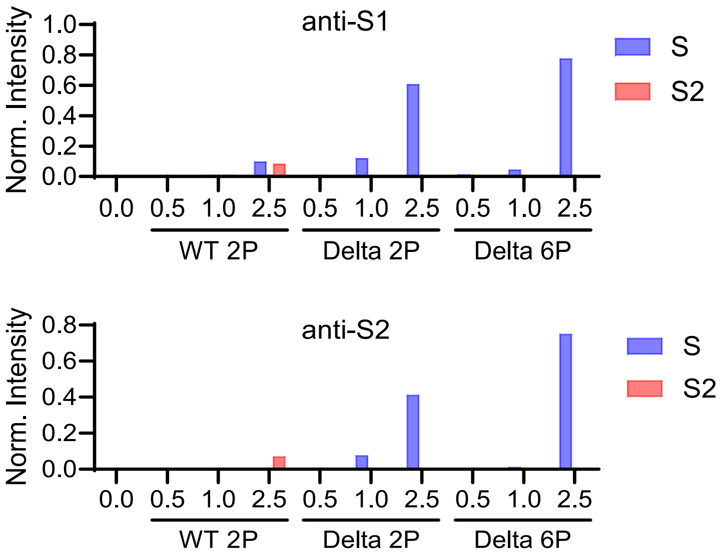

Supplementary Figure 2. Densitometric analysis of the Western blot shown in Figure 1A was performed using ImageJ. Band intensities were measured and normalized to beta-actin levels for consistency.

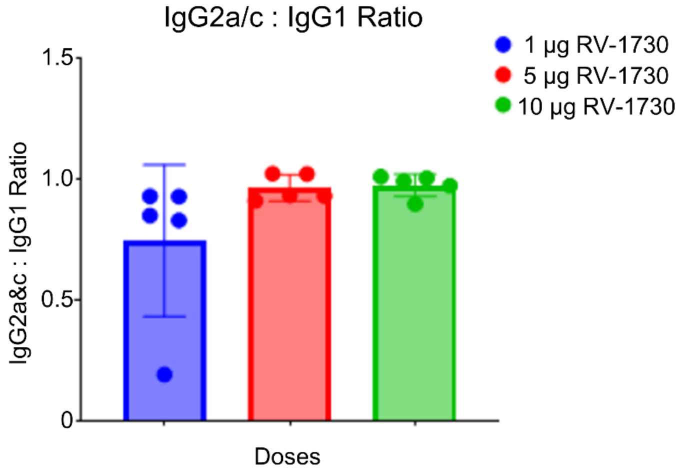

Supplementary Figure 3. Balb/c female mice were immunized at weeks 0 and 3 with 1, 5, 10 µg of RV-1730. Sera (n=5) were collected at 2 weeks post-boost and assessed by ELISA for SARS-CoV-2 Delta S-specific IgG1 and IgG2a/c. Endpoint titer ratios of IgG2a/c/IgG1 were calculated. Symbols are present individual mice.

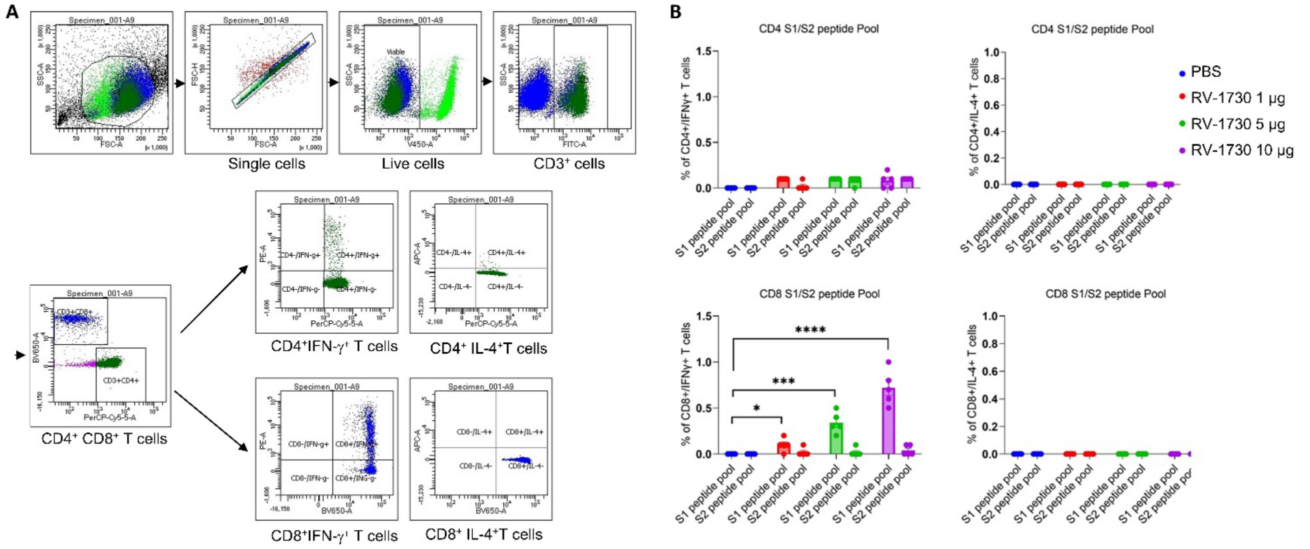

Supplementary Figure 4. Cytokine analysis of memory T cells using flow cytometry. Nine weeks post-boost, splenocytes were isolated from 5 mice per group and re-stimulated with no peptides or pools of overlapping peptides from SARS-CoV-2 S protein in the presence of a protein transport inhibitor cocktail. After 16 hours, intracellular cytokine staining (ICS) was performed to quantify $CD4^{+}$ and $CD8^{+}$ T cell responses. **A**. The gating strategy for assessing IFN-γ and IL-4 production in CD4+ and CD8+ T cells involved the following steps: Splenocytes were first gated from the total splenocytes population using forward scatter (FSC-A) and side scatter (SSC-A) axes. Outliers were excluded using FSC-W and SSC-W parameters. Cells positive for the viability stain were excluded. CD3+ cells were then gated based on CD4 and CD8 expression. Intracellular levels of IFN-γ and IL-4 were subsequently analyzed within the gated CD4+ and CD8+ T cell populations. **B**. Cytokine expression (IFN-γ and IL-4) in the presence of no peptides was considered background and subtracted from the responses measured from the S1 and S2 peptide pools for each individual mouse. *P<0.05, ***<0.001, and ****P<0.0001 (Student's t-test, unpaired).

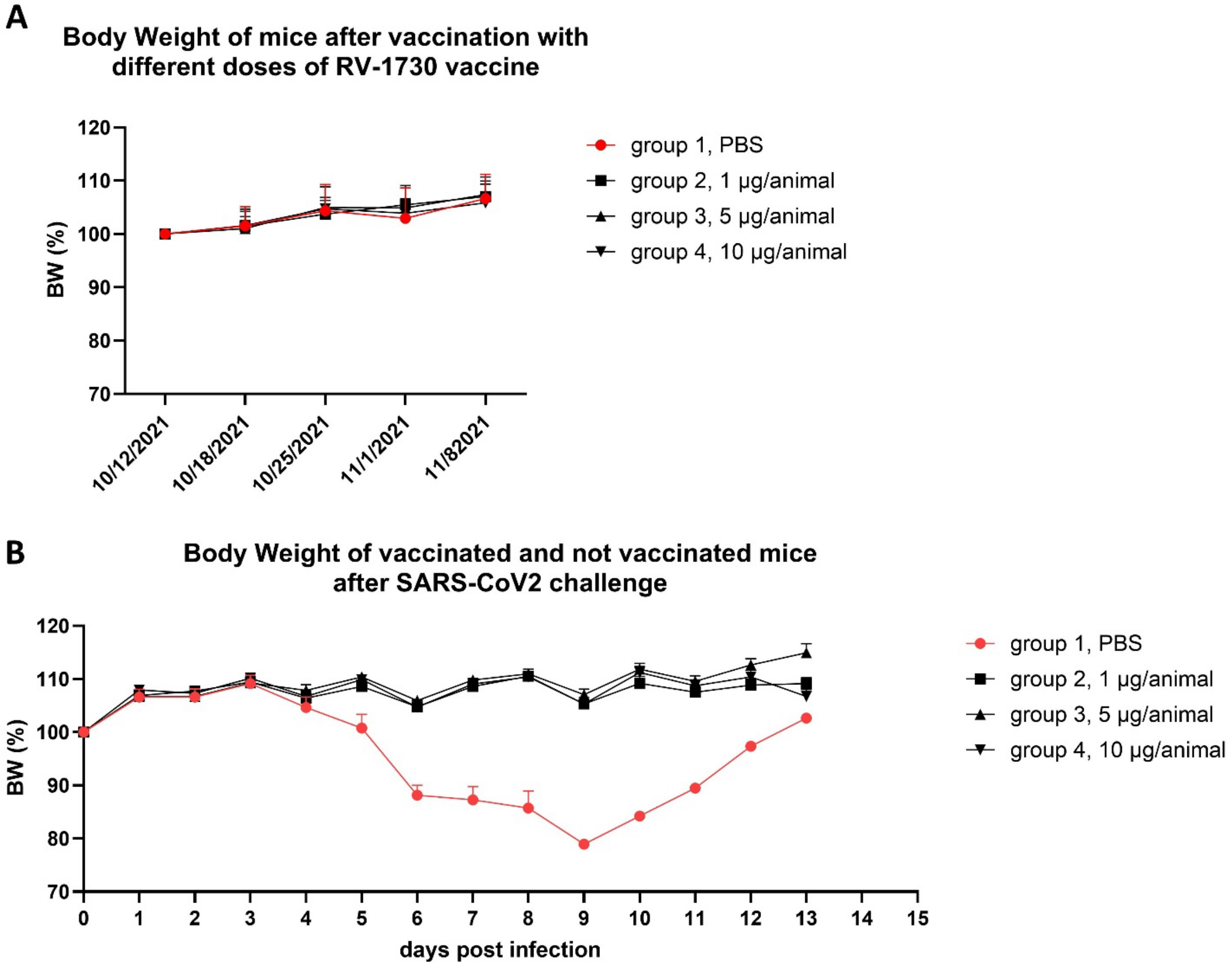

Supplementary Figure 5. Group mean body weights. A, B. Body weight (**A**) in mice of Groups 1 to 4 after immunization at NLS, and (**B**) after viral challenge with SARS-CoV-2/human/ITA/INMI1/2020 isolate at GMU. Body weight of each individual mouse was calculated as a percentage of body weight measured after delivery to NLS, and as a percentage of body weight measured before viral challenging at GMU. The mean and standard error of the mean (SEM) for each group of mice are presented.

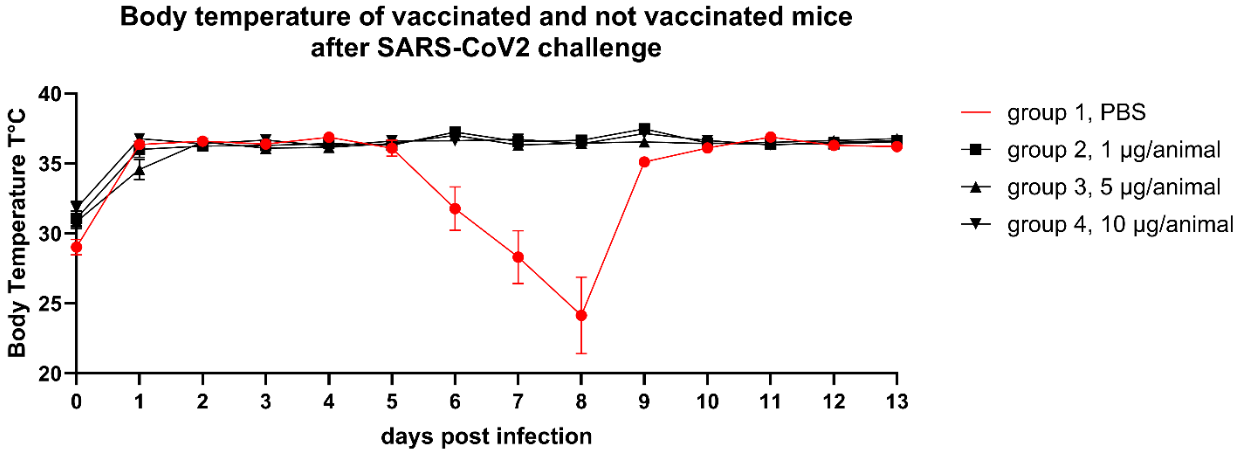

Supplementary Figure 6. Body temperature curves in mice of Groups 1 to 4 after viral challenge with SARS-CoV-2/human/ITA/INMI1/2020 isolated at GMU. The mean and standard error of the mean (SEM) for each group of mice are represented.

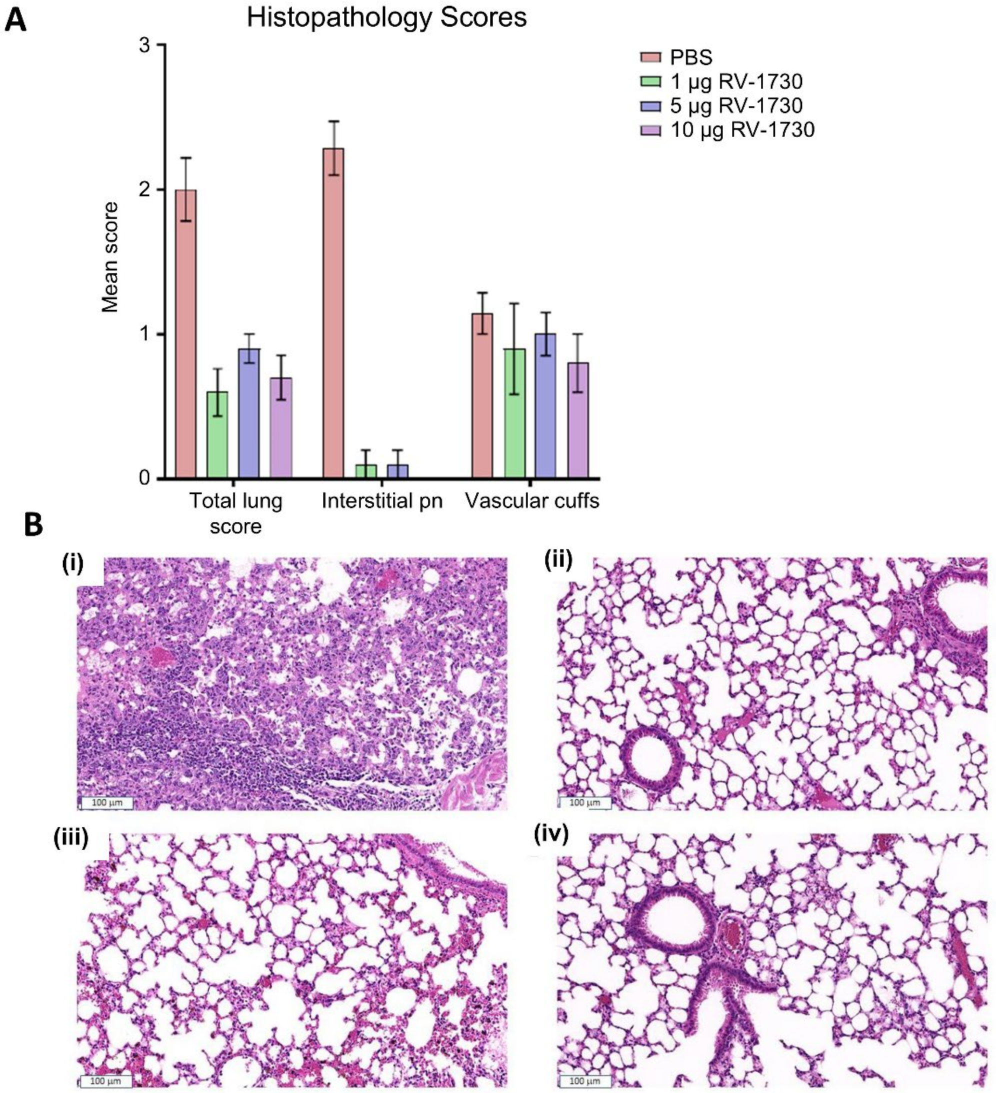

Supplementary Figure 7. Histopathology score for lung tissues in a K18-hACE2 mouse model of lethal infection. A. Histopathology score for lung tissues harvested at euthanasia**.** A graphical representation of the Total lung score, Interstitial pneumonia (pn), and Pervascular cuffs scored on a scale based on percentage of lung involved (0 = none; 1 = 0-25%; 2 = 26-50%; 3 = 51-75%; and 4 = 76-100%) and (specific lung lesions: 0 = none; 1 = minimal; 2 = mild; 3 = moderate; and 4 = severe) for each group is presented. **B.** Histopathology images for representative lung tissues harvested 13 days post-challenge with SARS-CoV-2. H&E-stained tissue sections from representative mice for each group were scanned at 20X magnification. (i) PBS control; (ii) 1 µg; (iii) 5 µg; (iv) 10 µg mouse. Scale bars (100 µm) are indicated at the left corner of each image.

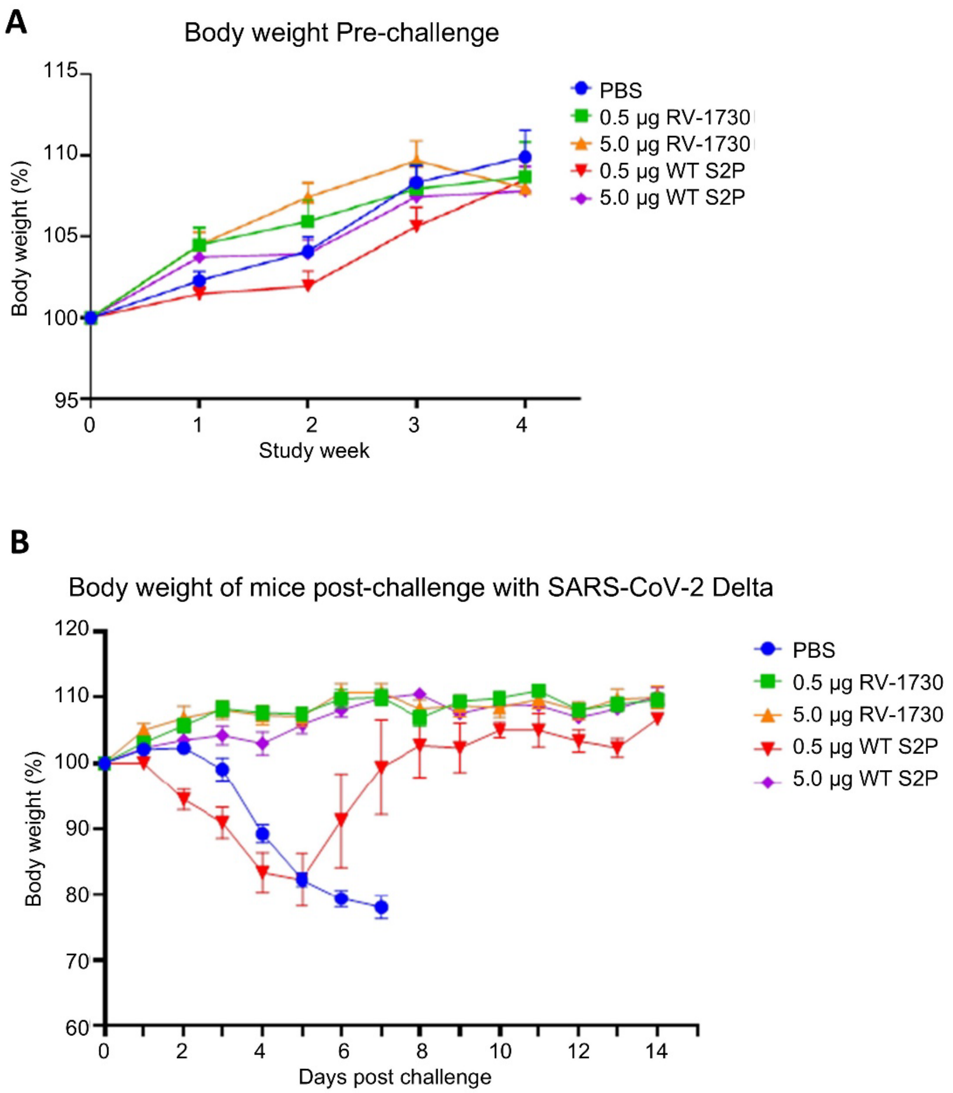

Supplementary Figure 8. Group Mean Body Weights. Body weight (**A**) in mice of Groups 1 to 5 after immunization at NLS, and (**B)** after viral challenge with SARS-CoV-2 B.1.1617.2 Delta variant at GMU. Body weight of an individual mouse was calculated as a percentage of body weight measured (**A**), and as a percentage of body weight measured before viral challenging at GMU (**B**). The mean and standard error of the mean (SEM) for each group of mice are presented.

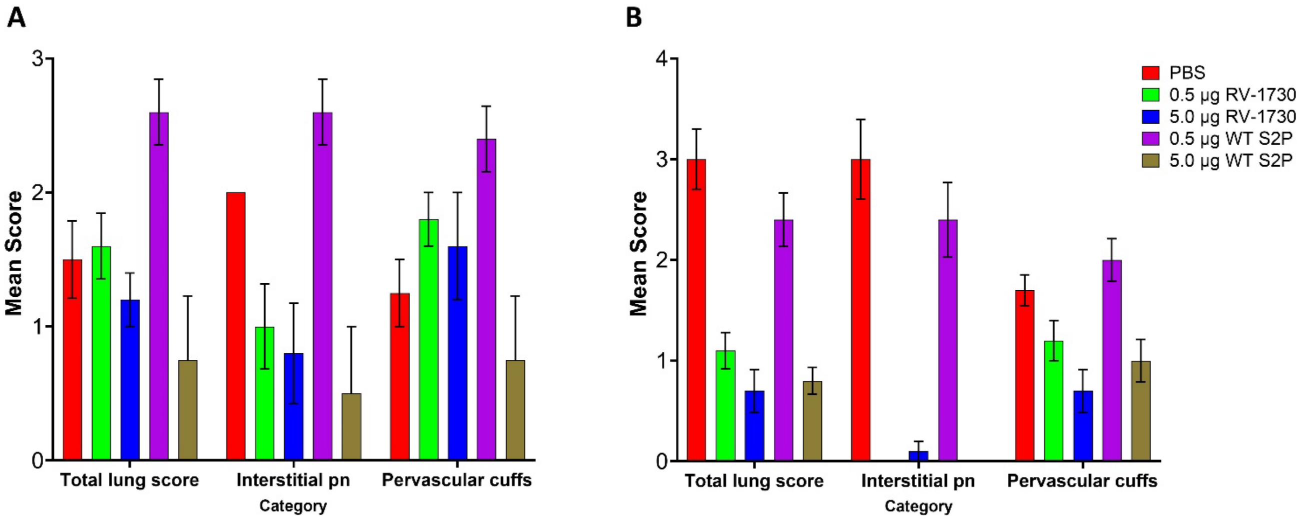

Supplementary Figure 9. Histopathology Score for Lung Tissues Harvested at Day 3 Post-challenge (A) and at Day of Euthanasia (B). A graphical representation of the Total lung score, Interstitial pneumonia (pn), and Pervascular cuffs scored on a scale based on percentage of lung involved (0 = none; 1 = 0-25%; 2 = 26-50%; 3 = 51-75%; and 4 = 76-100%) and (specific lung lesions: 0 = none; 1 = minimal; 2 = mild; 3 = moderate; and 4 = severe) for each group.

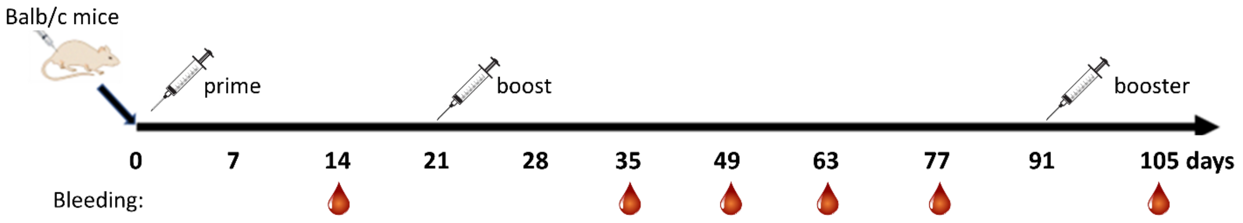

Primary vaccination schedule

Group	1st Immunization	2nd Immunization	Dose/Mice
Group 1	BNT162b2	BNT162b2	5 µg
Group 2	mRNA-1273	mRNA-1273	5 µg
Group 3	RV-1730	RV-1730	5 µg
Group 4	BNT162b2	RV-1730	5 µg
Group 5	mRNA-1273	RV-1730	5 µg

Booster vaccination schedule

Group	1st Immunization	2nd Immunization	3rd Booster	Booster dose
1	BNT162b2	BNT162b2	BNT162b2	2.5ug
2	mRNA-1273	mRNA-1273	mRNA-1273	2.5ug
3	RV-1730	RV-1730	RV-1730	2.5ug
4	BNT162b2	RV-1730	RV-1730	2.5ug
5	mRNA-1273	RV-1730	RV-1730	2.5ug

Supplementary Figure 10. Infographic to show vaccination timings and blood collection for homo/heterologous and booster. Female Balb/c mice received intramuscular injections of 5 µg each of BNT162b2, mRNA-1273, and RV-1730 on days 0 and 21 for as a prime-boost vaccination. Then, a booster dose of 2.5 µg for each vaccine was administered on day 72 post the first immunization. Blood samples were collected at 14, 35, 49, 63, 77, and 105 days after the initial immunization. Spleens were harvested at day 105 for T cell immunity experiments.

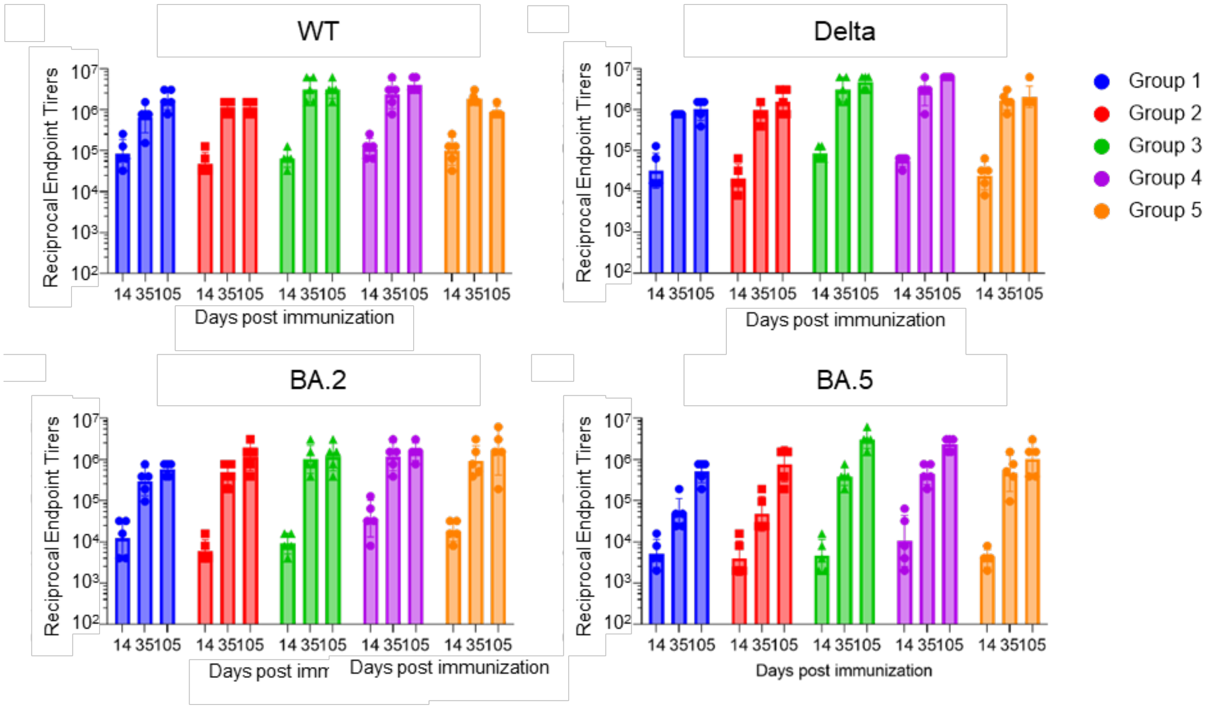

Supplementary Figure 11. Serological evaluation of the booster vaccination of commercial primary vaccines with RV-1730. Endpoint IgG titers against wild-type, Delta, Omicron BA.2, and BA.5 spike RBD proteins were assessed in sera collected on days 14, 35, and 105 from booster-immunized mice. Mice (n=5) were initially vaccinated with SARS-CoV-2 spike mRNA-LNP vaccines, as outlined in Supplementary Figure 10. Serum samples were analyzed for RBD-specific antibody levels using ELISA.

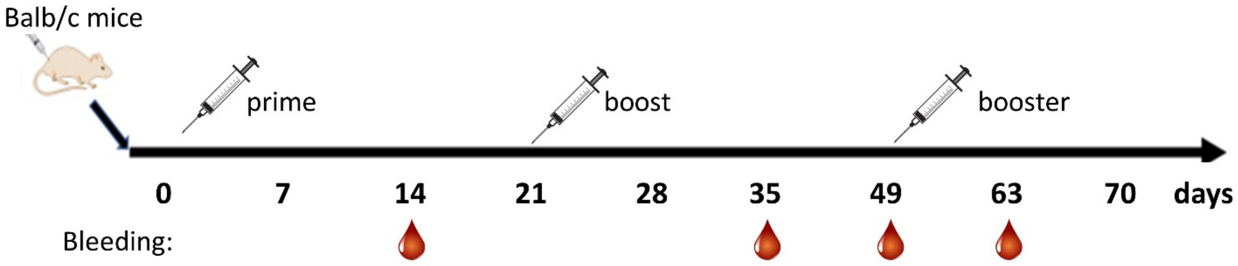

Group	1st Immunization	2nd Immunization	Bivalent Booster
1	PBS	PBS	PBS
2	BNT162b2	BNT162b2	BNT162b2 Original and Omicron BA.4/BA.5
3	BNT162b2	BNT162b2	RV-1731 (Delta and Omicron BA.1)
4	mRNA-1273	mRNA-1273	mRNA-1273 Original and Omicron BA.4/BA.5
5	mRNA-1273	mRNA-1273	RV-1731 (Delta and Omicron BA.1)
6	RV-1730	RV-1730	RV-1731 (Delta and Omicron BA.1)

Supplementary Figure 12. Infographic to show vaccination timings and blood collection for bivalent booster. Female BALB/c mice received intramuscular injections of 5 µg each of BNT162b2, mRNA-1273, and RV-1730 on days 0 and 21 for as a prime-boost vaccination. Then, a booster dose of 2.5 µg for each bivalent vaccine (BNT162b2 bivalent (Original and Omicron BA.4/BA.5), mRNA-1273 bivalent (Original and Omicron BA.4/BA.5) was administered on day 49 post the first immunization. Blood samples were collected at 14, 35, 49, and 63 days after the initial immunization. Spleens were harvested on day 70 for T cell immunity experiments.

A. Delta

DPI	Group 2	Group 3	Group 4	Group 5	Group 6
14	2.7×10^5	2.4×10^5	2.7×10^5	2.7×10^5	3.8×10^5
35	3.2×10^6	3.8×10^6	4.1×10^6	3.8×10^6	6.7×10^6
63	4.9×10^6	7.4×10^6	4.8×10^6	5.5×10^6	6.7×10^6

B. XBB1.5

DPI	Group 2	Group 3	Group 4	Group 5	Group 6
14	6.5×10^4	7.4×10^4	7.1×10^4	8.9×10^4	3.0×10^4
35	4.1×10^5	5.0×10^5	4.9×10^5	5.3×10^5	9.6×10^5
63	1.7×10^6	1.6×10^6	1.4×10^6	1.5×10^6	3.4×10^6

Supplementary Figure 13. Endpoint IgG titers of day 14, day 35 and post booster (day 63) SARS-CoV-2 spike mRNA-LNP immunized sera against Delta (A) and XBB1.5 variant spike RBD proteins (B). Mice (n=6) were prime immunized with monovalent SARS-CoV-2 spike mRNA-LNP (BNT162b2, mRNA-1273, and RV1731, 5 µg/mouse) and boost immunized with bivalent SARS-CoV-2 spike mRNA-LNP (BNT162b2, mRNA-1273, and RV1731, 2.5 µg/mouse) 3 weeks after prime-immunization, as depicted in Supplementary Fig. 11. Serum was collected to determine SARS-CoV-2 RBD protein-specific antibody levels by ELISA on day 14, one week before boost-immunization, and on day 35, two weeks after boost-immunization.

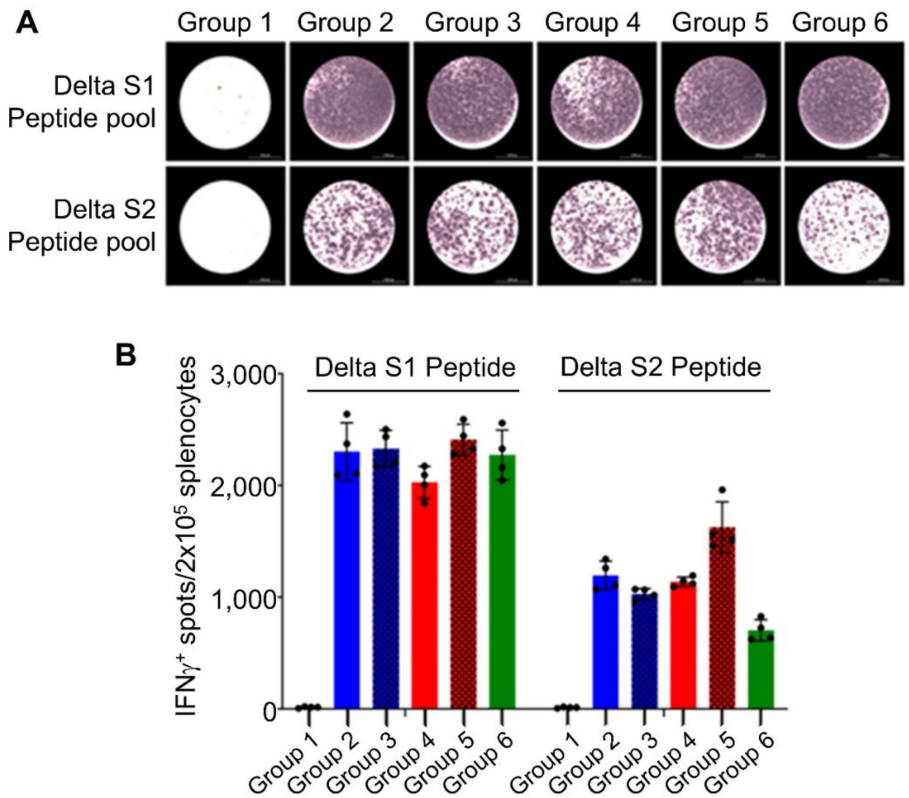

Supplementary Figure 14. IFN-γ ELISPOT analysis on splenocytes. The experiment was conducted as depicted in Fig. 10. Post booster shot, splenocytes were isolated from 5 mice per group, then splenocytes (2 x10^4 cells per 96 well) were plated onto mouse IFN-γ ELISpot plates (Mabtech) and re-stimulated ex vivo with pools of overlapping peptides from SARS-CoV-2 Delta Spike for 16 hours. Images were taken and quantified by using Cytation7. (**A**) Representative image of ELISpot. (**B**) Quantitative analysis of ELISpot.
